# Synergistic interaction of sprouting and intussusceptive angiogenesis during zebrafish caudal vein plexus development

**DOI:** 10.1038/s41598-018-27791-6

**Published:** 2018-06-29

**Authors:** Swapna karthik, Tijana Djukic, Jun-Dae Kim, Benoît Zuber, Andrew Makanya, Adolfo Odriozola, Ruslan Hlushchuk, Nenad Filipovic, Suk Won Jin, Valentin Djonov

**Affiliations:** 1 Institute of Anatomy, University of Bern, Bern, Switzerland; 2 Graduate School for Cellular and Biomedical Sciences, University of Bern, Bern, Switzerland; 3 Research and Development Center for Bioengineering, BioIRC, Sretenjskogustava 27, Kragujevac, Serbia; 4 Faculty of Mechanical Engineering, University of Kragujevac, SestreJanjic 6, 34000 Kragujevac, Serbia; 5 Yale Cardiovascular Research Center and Section of Cardiovascular Medicine, Department of Internal Medicine, Yale University School of Medicine, New Haven, CT 06511 USA; 6 School of Life Sciences and Cell Logistics Research Center, Gwangju Institute of Science and Technology, Gwangju, Korea; 7 Department of Veterinary Anatomy and Physiology, University of Nairobi, Nairobi, Kenya

**Keywords:** Imaging, Angiogenesis

## Abstract

Intussusceptive angiogenesis (IA) is a complementary method to sprouting angiogenesis (SA). The hallmark of IA is formation of trans-capillary tissue pillars, their fusion and remodeling of the vascular plexus. In this study, we investigate the formation of the zebrafish caudal vein plexus (CVP) in *Tg(fli1a:eGFP)*^*y7*^ and the synergistic interaction of IA and SA in crafting the archetypical angio-architecture of the CVP. Dynamic *in vivo* observations and quantitative analyses revealed that the primitive CVP during development was initiated through SA. Further vascular growth and remodeling occurred by IA. Intussusception contributed to the expansion of the CVP by formation of new pillars. Those pillars arose in front of the already existing ones; and in a subsequent step the serried pillars elongated and fused together. This resulted in segregation of larger vascular segments and remodelling of the disorganized vascular meshwork into hierarchical tree-like arrangement. Blood flow was the main driving force for IA, particularly shear stress geometry at the site of pillar formation and fusion. Computational simulations based on hemodynamics showed drop in shear stress levels at locations of new pillar formation, pillar elongation and fusion. Correlative 3D serial block face scanning electron microscopy confirmed the morphological substrate of the phenomena of the pillar formation observed *in vivo*. The data obtained demonstrates that after the sprouting phase and formation of the primitive capillary meshwork, the hemodynamic conditions enhance intussusceptive segregation of hierarchical vascular tree i.e. intussusceptive arborization resulting in complex vascular structures with specific angio-architecture.

## Introduction

Angiogenesis is the process of developing new blood vessels in physiological and in many pathological conditions. It comprises two discrete types, sprouting angiogenesis (SA) and intussusceptive angiogenesis (IA). Expansion of capillary networks by a phenomenon called “growth within itself” was termed intussusceptive microvascular growth (IMG). IA is also known as splitting angiogenesis or non-sprouting angiogenesis or inverse sprouting angiogenesis; it is characterized by formation of transluminal tissue pillars and subsequent vascular splitting. These trans-capillary or intussusceptive pillars are formed by protrusion of opposing capillary endothelial cells into the capillary lumen thus creating a zone of contact, leading to perforation of the bilayer of endothelial cells and increase in girth of the newly formed interstitial pillar due to invasion by fibroblasts and pericytes^1–5^.

The primitive capillary plexus formed by vasculogenesis, sprouting or intussusception is a finally mesh-like structure, i.e. consists of multiple pillars of different sizes. The subsequent growth and remodelling are achieved through different forms of IA, namely: Intussusceptive microvascular growth (IMG), Intussusceptive arborisation (IAR) and Intussusceptive branching remodeling (IBR). Intussusceptive microvascular growth reflects initiation of pillar formation and their subsequent expansion with the result that the capillary surface area is greatly enhanced, but the capillary plexus remains primitive and disorganized. In contrast, intussusceptive arborization entails formation and subsequent fusion of serried pillars delineating straight capillary segments and generations of smaller future feeding and draining microvessels. By this process, fusing pillars allocate hierarchical microvascular segments restructuring the primitive capillary meshwork to a vascular tree. Optimization of local vascular branching geometry occurs through intussusceptive branching remodelling, so that the vasculature is remodelled to meet the local hemodynamic demand. The latter process occurs via transluminal pillars that are formed close to arterial or venous bifurcation sites. Enlargement of such pillars, their subsequent fusion with the connective tissue at the bifurcation narrows the bifurcation angle. Eccentric formation and expansion of pillars at the bifurcation often results in a sub-form of IBR, namely intussusceptive vascular pruning^2,3,6–8^. In the strict sense, only IMG could be considered as an angiogenic mechanism due to the fact that it creats new vascular entities. IAR and IBR are important in creation of the local organ-specific angio-architecture as they are associated mainly with reduction in the vascular complexity and pruning could be considered for this reason, as pruning is defined as reduction of the vascular complexity. Under physiological conditions, three facets of IA (IMG; IAR; IBR) work hand in hand and are essential processes for the formation and restoration of an organ-specific angio-architecture. IA has been studied in several experimental models and was first described in vessels of the developing rat lung^9^ and was later demonstrated in other organs including the chicken chorio-allantoic membrane (CAM)^10^, in the developing chicken choroid vasculature^11^, metanephric avian kidney^12^ and chicken embryo lung^13^. IA also occurs in different pathological situations like tumor growth, murine colitis, and in neurotoxic disease^14^.

Although the morphological aspects of IA have been described in our previous works, unlike SA the molecular mechanisms of IA are yet to be investigated. Hemodynamic parameters of blood vessels such as wall shear stress and red blood cell (RBC) velocity are known to be the key modulators in the process of angiogenesis. One of the main differences between sprouting and intussusceptive angiogenesis is that sprouts appear (initially) in regions lacking perfusion, whereas pillars are formed in the perfused vascular areas^15–20^. These observations clearly point towards a significant role of blood flow and shear stress mediating intussusceptive vessel splitting. However, the molecular link between shear stress and its effects on regulating intussusceptive angiogenesis are not well understood. To this date, there is no single mechanotransducer clearly linked to IA, but several mechanosensing biological components, which could potentially regulate blood flow-driven pillar formation, have been documented^21^.

The synergistic interaction between IA and SA during development is largely unknown. We utilized zebrafish CVP as a model to better understand the synergistic interaction between IA and SA. CVP is mainly to study venous angiogenesis and how this plexus remodels gradually and simplifies to become a single vascular tube. In this study by employing the developing zebrafish caudal vein plexus (CVP) we have identified intussusceptive remodeling and shown its response to blood flow alterations. On the basis of our findings, we hypothesize that intussusceptive angiogenesis is promoted and governed/regulated by the specific hemodynamic profile. We show that intussusception is responsible for capillary expansion and in a subsequent step to vascular remodeling and intussusceptive arborization forming in this way the hierarchical angio-architecture of the CVP.

## Materials and Methods

### Zebrafish Maintenance

*Tg(fli1a:eGFP)*^*y7*^;*Tg(kdrl:EGFP)*^*s843*^ transgenic zebrafish lines were used throughout this study. Genetically modified embryos were obtained from naturally spawning transgenic lines and staged according to Kimmel *et al*.^22^. Zebrafishes (*Danio rerio*) were raised in a dedicated zebrafish facility with the system water maintained at 28.5 °C with 14 h light 10 h darkness diurnal rhythm. Adult male and female zebrafishes were separated for a week prior to breeding. Subsequently they were put together at male to female ratio of 2:1. The embryos were maintained in standard embryo medium (1 × E3 medium) throughout the experiments. The transgenic lines were obtained from aquatic resource program (Children’s Hospital, Boston, USA). All the animal experiments were performed according to the guidelines of Swiss animal welfare act (license number is BE413). According to the Swiss government guidelines, experiments based on zebrafish embryos aged less than 48 hours of post fertilized embryos are exempted from animal permission.

### Intravital microscopy and live confocal microscopy

*Tg(fli1a:eGFP)*^*y7*^ transgenic zebrafish embryos were incubated at 28.5 °C until 24 h post fertilization (hpf). Embryos were screened for GFP expression and the positive ones were treated with 0.003% 1-phenyl-2-thiourea (PTU) solution in E3 medium to prevent pigment formation at 24 hpf. The chorions were removed and mounted on low melting point agarose gel to enable imaging of CVP formation. Establishment of the vascular pattern and blood flow were monitored between 24 – 42 hpf by fluorescence stereomicroscopy (Leica stereomicroscope M205FA, Leica microsystems, Switzerland). Still images were captured and blood flow was recorded as video files using Leica camera (DFC365X) and software (Leica AF600). These videos were processed further for morphometric quantifications and simulations.

Dechorionated embryos were mounted on 0.4% of low melting point agarose gel containing 0.01% of tricaine in embryos medium (standard E3 medium). Time-lapse images were recorded in Axiovert 200 M microscope with laser scanning module LSM 5 Duo live (Zeiss, Germany) using 20X objective between 24 and 40 hpf, and z-stacks were also performed. Z-stack image projections were processed using Imaris software (v7.7.2, Bitplane AG, Switzerland) for 3-dimensional (3D) visualization of pillars in the CVP.

### Morphometric analysis of the caudal vein plexus

The prevalent angiogenic mode (sprouting vs. intussusception) was tightly monitored by *in vivo* observation with emphasis on the CVP starting from 24 hpf up to 42 hpf. The images acquired were used to quantify sprouts and pillars. Pillars were identified as dark holes in the green vascular plexus with diameters roughly ≤ 2.5 µm. All holes greater than 2.5 µm were considered to be meshes (large pillars). A borderline between perfused and the non-perfused area in the CVP was drawn with the help of blood flow videos captured along the still pictures. The following parameters were calculated between perfused and non-perfused areas using Cell^D software (Olympus soft imaging solutions GmbH, Germany)

### Vessel area

Vessel area (VA) was obtained as the ratio of total number of points falling on the vascular (green) surfaces, [Pp(Vs)] and the point-associated area in µm^2^ [Pp(A)]. Thus; VA = Pp(Vs) * Pp(A).

### Numerical pillar density

Numerical density of the pillars [NP(Pr,Vs)] was estimated as the total number of pillars counted per µm^2^ of vessel area, NA(Pr). Thus; NP(Pr,Vs) = NA(Pr)/VA.

### Numerical sprout density

Numerical density of the sprouts [NS(Spr,Vs)] was calculated as the total number of sprouts counted per µm^2^ of vessel area. NA(Spr). Thus; NS(Spr,Vs) = NA(Spr)/VA.

### Computational simulation and software details

Experimental images were used to define the geometry of the domain. Pillar positions and dimensions were manually highlighted in these images. The obtained information about geometry was then used to create the finite element mesh of the model. Finite element mesh was generated using FEMAP software version 10 (Siemens PLM Software, Piano, TX, USA), and was paired with our in-house developed software tool written in C++. This software tool is used to adapt the finite elements mesh to the format that is appropriate for the numerical simulations. The in-house developed software package PakF^23,24^ was already successfully applied to model blood flow through blood vessels in chick embryos and to analyze the creation of pillars^25^. This method is used in this paper to simulate blood flow through the caudal artery and caudal vein of the zebrafish. In numerical simulations the units for physical dimensions of the domain are micrometers, obtained velocity distribution is shown in micrometers/second, obtained pressure and wall shear stress distributions are shown in Pascal (N/m2). Characteristics of blood are defined as follows: density is set to 1.05 g/cm^3^ and dynamic viscosity is set to 3.675·10^−3^ Pa·s. For detailed description of the simulation calculation, please see supplementary data Materials and Methods.

### Drug treatments

*Tg(fli1a:eGFP)y*^7^ embryos were maintained in E3 medium until 26 hpf and the CVP was imaged using epifluorescent microscope. Control groups and treated groups were separated accordingly. Freshly prepared isoprenaline hydrochloride (50 µM, Sigma-Aldrich GmbH, Switzerland) and 2,3 BDM (6 mM, Sigma-Aldrich GmbH, Switzerland) was added to E3 medium of the treated embryos at 26 hpf and 24 hpf respectively. The embryos were incubated at 28.5 °C until 30 hpf. The control embryos in E3 medium were maintained in the same incubator until 30 hpf. The drug solution for the treated group was replaced every 45 min with freshly prepared drug solution and incubated at 28.5 °C until 36 hpf. Images of the CVP from both the control and treated groups were obtained.

### Injection of antisense morpholino oligonucleotide (MO)

Control, *troponin 2a*, (*tnnt2a)*^26^ and *gridlock (grl)*^27^ MOs were purchased from Gene Tools, LLC, USA. MOs were injected with phenol-red (0.05%) in zebrafish embryos at the 1~2 cell stage at a concentration of 3-5 ng/embryo. The MOs of the following sequences were used:

Control MO; 5′-CCTCTTACCTCAGTTACAATTTATA-3′,

*tnnt2a*MO; 5′-CATGTTTGCTCTGATCTGACACGCA-3′,

*grlMO*; 5′-CGCGCAGGTACAGACACCAAAAACT-3′,

Embryos were treated with 0.003% PTU solution to prevent pigment formation at 24 hpf and then mounted using a 1% low-melting agarose on the glass bottom dish and laterally oriented. CVP in the tail region (within somite boundary 16 and 21) were imaged and processed using a Nikon confocal microscope (Nikon Instruments Europe B.V, Switzerland) and Velocity program (PerkinElmer, Inc., USA). ImageJ and Fiji programs were used to create maximum intensity projections and depth coding of Z-stack images.

### Three-dimensional serial block face scanning electron microscopy

The *Tg(fli1a:eGFP)*^*y7*^ embryos were imaged and fixed in karnovsky solution (2.5% glutaraldehyde, 2.5% formaldehyde, 0.1 M cacodylate pH 7.4) overnight at 4 °C. Further, the embryos were washed 5 times for 3 min each in 0.15 M cacodylate buffer. Embryos were then incubated in 0.15 M cacodylate solution containing 1.5% potassium ferrocyanide and 2% aqueous osmium tetroxide (Electron microscopy sciences) for 1 hour at room temperature. The samples were washed in water and incubated in 1% filtered thiocarbohydrazide (Sigma-Aldrich GmbH, Switzerland) solution for 20 min at room temperature. Subsequently, they were washed gently 5 times for 3 min in milliQ water at room temperature and then incubated in 2% aqueous osmium tetroxide for 30 min at room temperature. Following this step, the embryos were washed again 5 times for 3 mins in milliQ water and then incubated in 1% uranyl acetate and left at 4 °C overnight. The following day the embryos were washed again 5 times for 3 min in milliQ water at room temperature and incubated in Walton’s lead aspartate solution^28^ at 60 °C for 30 min. Then they were washed in milliQ water 5 times for 3 min and dehydrated by immersion in ascending concentrations of ethanol starting from 20%, then 50%, 70%, 90%, and 100%, 5 min each at room temperature. After the final incubation in ethanol, the embryos were transferred again to pure ethanol for 10 min at room temperature. Finally the embryos were placed in mixtures of Durcurpan (Sigma) and ethanol as follows: 25% Durcupan for 2 hours, then 50% Durcupan for 2 h, and 75% Durcupan for 2 hours. Embryos were then placed in 100% Durcupan overnight and then transferred into fresh 100% Durcupan for polymerization in silicon molds at 60 °C for 48 h.

The blocks were trimmed and transverse sections were cut through the trunk of the zebrafish embryos. To perform serial block face scanning electron microscopy (SBF-SEM), ultra-thin sections were imaged with a Quanta 250 FEG (FEI) scanning electron microscope equipped with a 3View2.XP *in situ* ultramicrotome (Gatan). The block face images obtained were taken with 3 kV acceleration voltage and at 10 Pa pressure (low vacuum). Each slice was 75 nm thick and imaged with a magnification of 626.0×, pixel size of 21 nm and pixel dwell time of 1 µs (min frame time (s) = 34.169). The images were further processed for 3D reconstruction using Imaris software (Ver.7.7.2).

### Statistical analysis

The data obtained were compared by non-parametric Mann-Whitney statistical test using the GraphPad Prism software (version 5.0; San Diego, CA). Data were presented as mean ± SD and asterisks were used to indicate significant differences (*p < 0.05; **p < 0.01; and ***p < 0.005).

## Results

### Morphogenesis of zebrafish caudal vein plexus (CVP)

**Figure 1:**
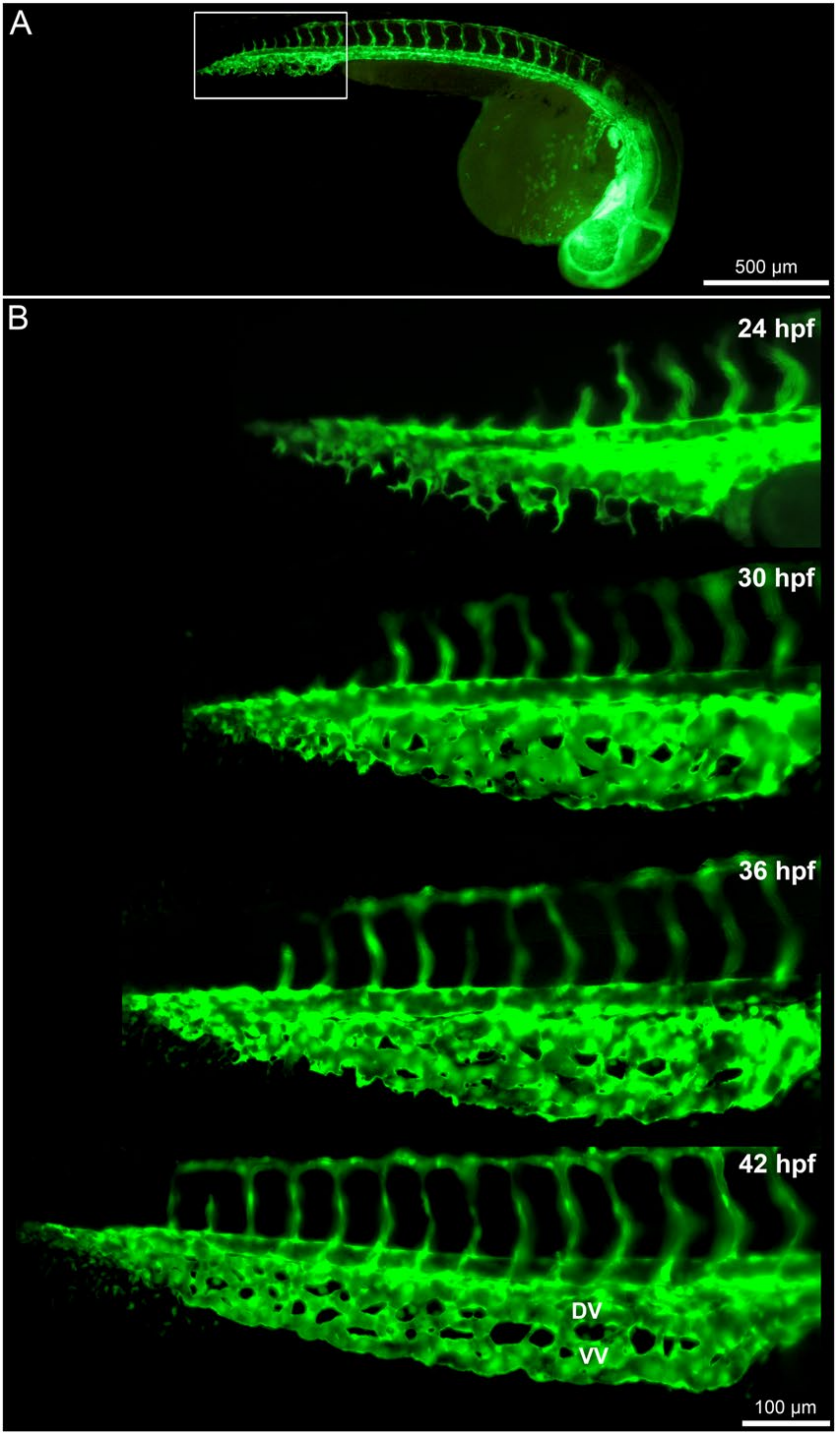
Time lapse *in vivo* images of the caudal vein plexus (CVP) from 24-42 hpf. (**A**) Overview of the blood vessels in *Tg(fli1a:eGFP)*^*y7*^ of the whole zebrafish embryo with the highlighted region (white box) showing the developing CVP. (**B**) *In vivo* still images of the CVP from selected ages between 24 hpf and 42 hpf indicating the mode of angiogenesis. Sprouting and anastomoses are evident at 24 hpf while pillar formation starts from 30 hpf increasing in intensity towards 42 hpf.

**Figure 2:**
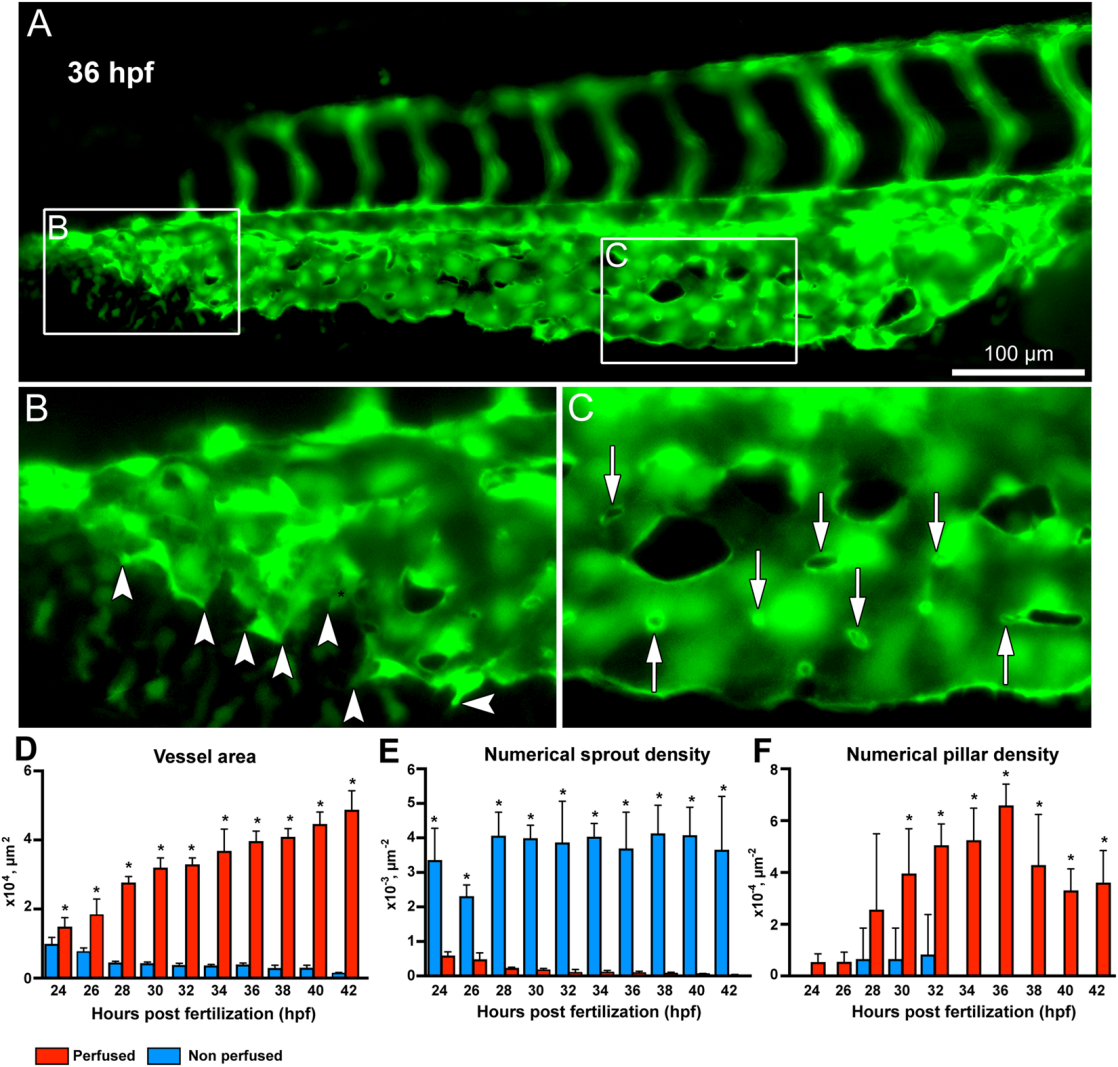
Morphometric analysis of sprouting vs.intussusceptive angiogenesis between perfused and non-perfused regions of the zebrafish CVP. (**A**) At 36 hpf the zebrafish embryo shows both intussusceptive and sprouting angiogenesis. (**B**) The box B in the distal region of the CVP is marked to show the sprouts in the non-perfused region with the arrowheads pointing to the corresponding enlarged image. (**C**) The box C in the proximal region of the CVP shows the pillars in the perfused region marked with white arrows in the corresponding enlarged image. (**D**) The bar graph represents the vessel area (VA) of the zebrafish CVP, the VA in the perfused region is significantly (p < 0.05) increased in comparison with the non-perfused region. (**E**) The numerical sprout density (i.e. number of sprouts/vessel area) is significantly (p < 0.05) increased in the non-perfused region compared to the perfused region. (**F**) While the numerical pillar density (i.e. number of pillars/vessel area) increases with time reaching the maximum pillar density at 36 hpf and subsequently decreases due to pillar fusion and splitting, the values represented in the graph are mean ± SD (n = 4). Asterisks (*) indicate significant increase.

An overview of the developing CVP of *Tg(fli1a:eGFP)*^*y7*^ zebrafish embryos between 24 hpf and 42 hpf is presented in Fig. 1A. With the initiation of blood circulation after 24 hpf, numerous angiogenic sprouts propagated predominantly towards the avascular microenvironment of the CVP and these angiogenic sprouts later interconnected each other to form the primitive CVP (Figs 1B and 2A, B). Lack of vascular perfusion was observed in non-lumenized areas of sprouts during the early development of CVP. Increase in area of perfusion following maturation and vascular lumenization of the developing capillary plexus were observed from 30 hpf onwards (Fig. 1B and Movie S1). After 36 hpf, expansion of the CVP was mainly driven by formation of intussusceptive pillars, which were mostly observed in the proximal perfused region (Fig. 2C) while the angiogenic sprouts drove vascular growth in the distal non-perfused region of the CVP (Fig. 2B). The transluminal intussusceptive pillars were identified as incipient tiny holes in the CVP with a diameter ≤ 2.5 µm. Historical as a transluminal pillar with endothelial columns with a diameter less than 2.5 µm has been defined. Certainly, the diameter could be variable. The appearance of tiny holes was clearly documented.

### Morphometric analysis of zebrafish CVP

**Figure 3:**
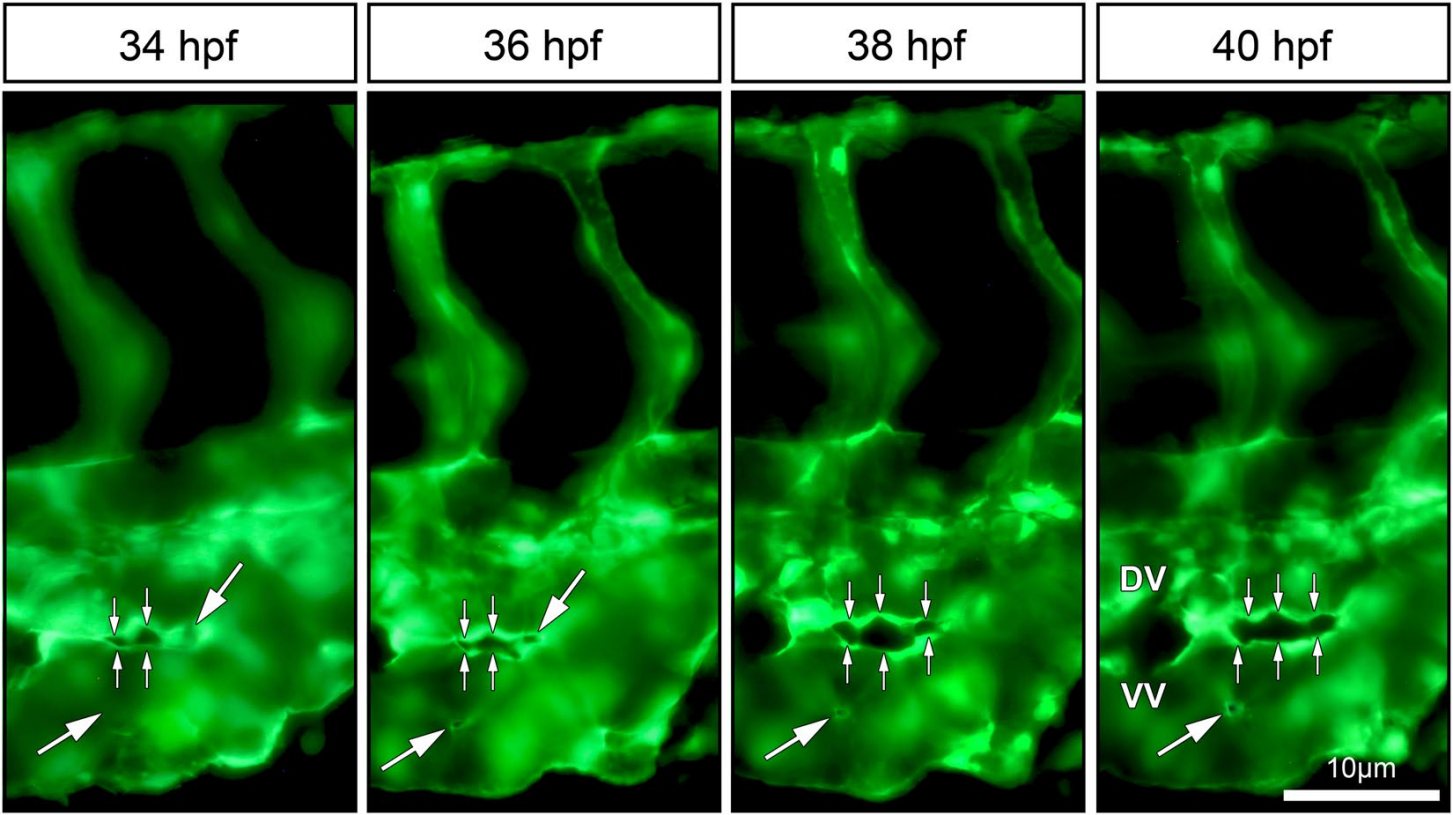
*In vivo* imaging of intussusceptive pillar formation followed by fusion and splitting in the CVP of zebrafish embryo. The large white arrows represent a clear vessel at 34 hpf and newly formed pillars (appearing as a tiny holes) in the same region of observation at 36 hpf. The small white arrows show pillar fusion and splitting of the dorsal (DV) from the ventral (VV) vein in between from 36-40 hpf. For further information, see Movie S2.

The perfused and non-perfused regions of CVP were delineated for the morphometric analysis to calculate vessel area, numerical sprout density and numerical pillar density. In line with our expectations, the vessel area of the perfused region between 24 to 42 hpf increased significantly from 1.4 ± 0.1 × 10^4^ µm^2^ to 4.8 ± 0.3 × 10^4^ µm^2^ in the developing CVP. In contrast, the vessel area of the non-perfused region decreased from 0.9 ± 0.1 × 10^4^ µm^2^ (at 24 hpf) to 0.1 ± 0.01 × 10^4^ µm^2^ (at 42 hpf) in the distal region of the growing CVP (Fig. 2D). The average numerical sprout density of the non-perfused region was 3.7 ± 0.1 × 10^−3^ µm^−2^ for all time points between 24 hpf and 42 hpf; this remained significantly higher when compared to the sprout density of the perfused regions of the CVP (Fig. 2E). Sprouting was the dominant mode of vessel growth at all time points between 24 hpf–42 hpf in the non-perfused region. On the other hand, the numerical pillar density increased significantly from 3.9 ± 0.8 × 10^−4^ µm^−2^ at 30 hpf in the perfused regions and it reached the highest density 6.5 ± 0.4 × 10^−4^ µm^−2^ at 36 hpf (Fig. 2F). Subsequently, after 36 hpf the numerical pillar density decreased to 3.5 ± 0.6 × 10^−4^ µm^−2^ at 42 hpf due to pillar fusion, reshaping and splitting of the capillary plexus in the median plane of the vascular network. The remodeling of the CVP ultimately led to maturation of the vascular network by segregation of the vascular entities into a distinct dorsal vein (DV) and a ventral vein (VV) (Figs 1 and 3). These results clearly underpin the process between sprouting and intussusceptive angiogenesis during early embryonic development of the zebrafish CVP.

### Intussusceptive pillar formation *in vivo* and its 3D structure

Transluminal intussusceptive pillar formation is a dynamic and sequential process leading to IA. The progression of emerging pillars and remodeling of the developing capillary plexus in *Tg(fli1a:eGFP)*^*y7*^ zebrafish embryos, were observed by series of high magnification *in vivo* fluorescence images and real time videos of blood flow in the CVP. The precise location of pillar appearance in the vascular network cannot be predicted in advance. Tiny pillars with a diameter ≤ 2.5 µm appeared after 32 hpf of CVP development in the well-perfused regions of the capillary plexus. Subsequently, we made an effort to track the pillar formation along with pillar fusion and splitting of vessels during development of the CVP in the same embryo at different time points. At 34 hpf, (Fig. 3) the long arrows point at a clear vessel without any visible structural change on the top of the vessel. Apparently, within 2 hours of monitoring, a tiny hole appeared in the capillary, which is pinched in the center of the same region of observation. The captured blood flow videos depicted pillar formation along with bouncing of RBCs in and around the pillar formation region (Movie S2). Consecutively, at 38 hpf, pillar reshapes and increases in its girth along with the growth which is much evident and proves that the pillar is formed in the perfused region (Movie S2). Interestingly, the short arrows of the Fig. 3 at 34 hpf depict how a newly formed pillar fuses with the adjacent pillars congruently by 36 hpf and splits the vessel with time by increase in intercapillary space that leads to the formation of future DV and VV. The obtained z-stack confocal images were reconstructed for 3-dimentional visualization of the pillar (Movie S3).Well-established intraluminal connection of endothelial cells is visualized in the reconstructed intussusceptive pillar in the capillary network. This strongly suggests that the tiny holes appearing on the surface of the capillary reflect the emergence of transluminal intussusceptive pillar. These insights of pillar formation further elicited for the correlative study between *in vivo* fluorescent imaging and its corresponding ultra-structural analysis.

### Serial block-face scanning electron microscopy (SBF-SEM) of intussusceptive pillars in CVP

**Figure 4:**
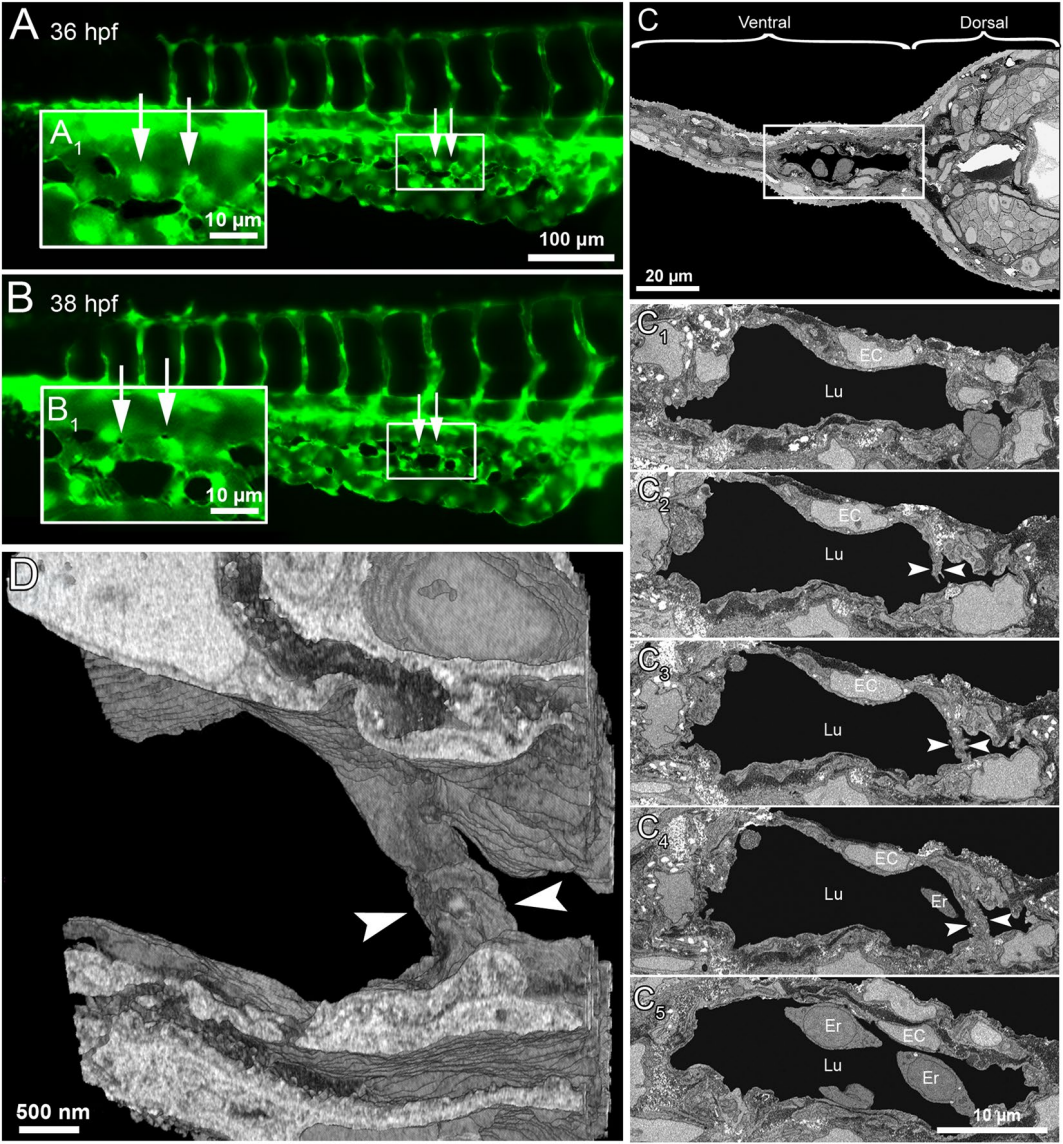
Serial block face electron microscopy (SBF-EM) demonstrating the 3D ultra-structure of newly formed intussusceptive pillar. (**A**) *In vivo* GFP images of CVP at 36 hpf, revealing intact vascular surface; the site of future pillar formation is indicated by white arrows. (**A**_**1**_) Symbolizes the rectangle in A at higher magnification. (**B,B**_**1**_) At 38 hpf in the area of interest dark dots (indicated by arrows) represent the newly-formed pillars. (**C**) Overview of the SBF-SEM transverse section of zebrafish embryo (each slice measuring 75 nm thick) shows the dorsal and ventral regions at 38 hpf and the ventral region with the CVP is indicated in the white rectangle. (**C**_**1**_**–C**_**4**_**)** Corresponding to CVP serial sections (650, 677, 682, 690 and 693) illustrating the ultra-structure of a trans-luminal intussusceptive pillar (indicated by arrowheads). The other structures in the CVP like endothelial cells (EC), erythrocytes (Er) and vascular lumen (Lu) are respectively indicated. (**D**) Represents the 3D reconstruction of intussusceptive pillar (indicated with large arrowheads) corresponding to the proximal one in B_1_ and to the C1–C4 SBF-SEM sections. For further information, see supplementary Movie S4.

**Figure 5:**
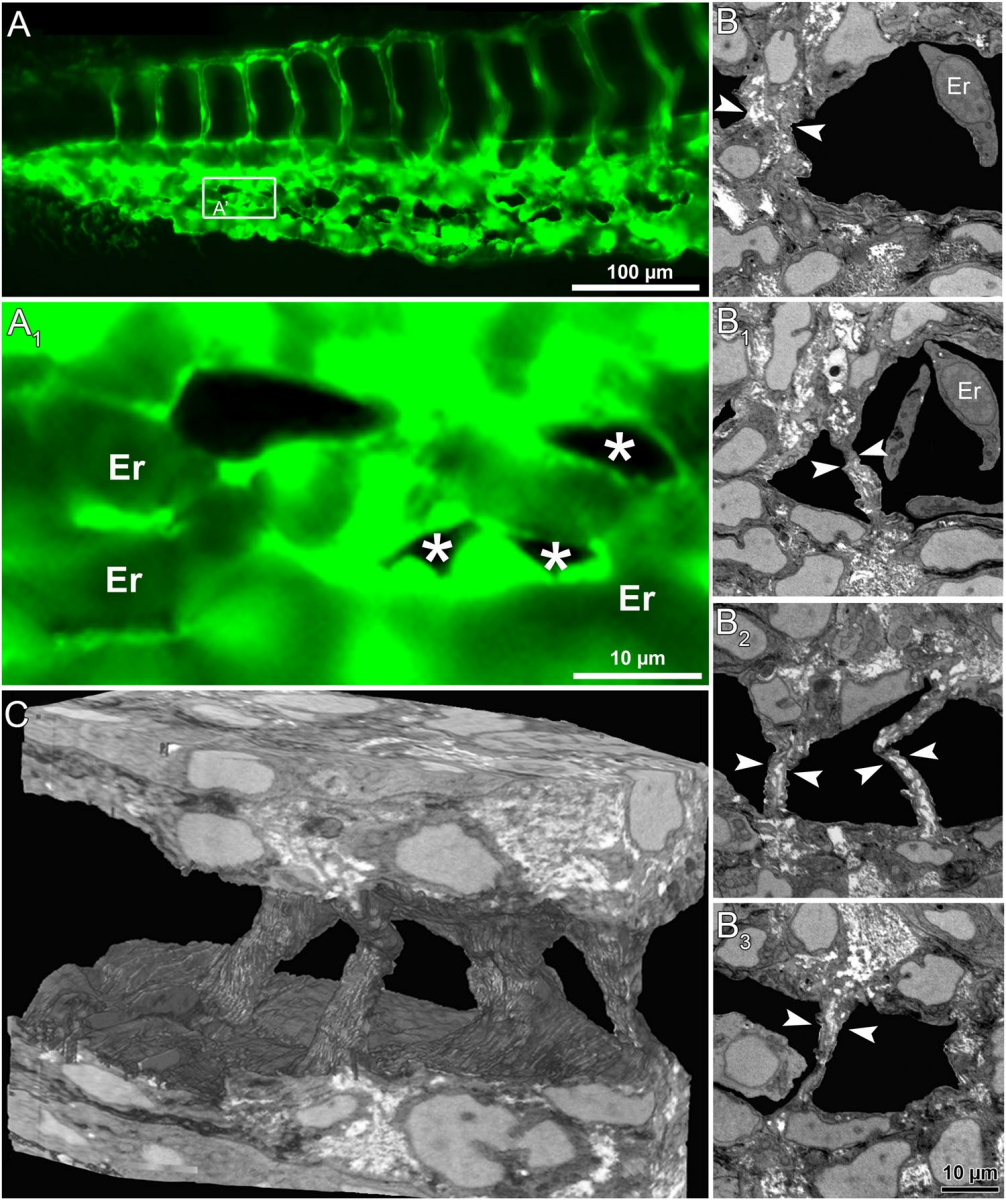
*In vivo* images and serial block face electron microscopy (SBF-SEM) sections obtained during IA mediated remodeling of zebrafish CVP. (**A**) *In vivo* images of CVP from zebrafish embryo and the corresponding region of interest (**A**_**1**_) in the GFP image of intussusceptive pillars (indicated by asterisk) shown at higher magnification. (B-B_3_) Representative SBF-SEM images showing a set of 4 pillars found in the distal region of the remodeling CVP (series of sections comprising 500 slices, each with 50 nm thickness). (**C**) Three dimensional reconstruction of SBF-SEM sections illustrates the volume of intussusceptive pillars. Erythrocytes have been removed in order to demonstrate the spatial orientation of three pillars. For further information, see Supplementary Movies S5 and S6.

The ultra-structural details of the trans-luminal intussusceptive pillars in the zebrafish embryos were investigated using serial block face scanning electron microscopy (SBF-EM). The CVP development of *Tg(fli1a:eGFP)*^*y7*^ zebrafish embryos was imaged at 38 hpf (Fig. 4A_1_ and B_1_). A dataset containing approximately 5000 serial transverse sections was scanned at voxel resolution of 21 × 21 × 50 nm from the distal part of the extended yolk sac region through the entire caudal region of the embryo. From the datasets obtained, the dorsal and ventral parts of the zebrafish embryo (Fig. 4C) could be differentiated. As the region of interest was CVP, the entire focus moved towards the ventral regions. The CVP lumen (Lu) was identified while the intermittent erythrocytes (Er) were distinguished with the help of their prominent nuclei. The endothelial cells (EC) lining the lumen and their junctions were visible. The trans-luminal intussusceptive pillars usually appear across the lumen comprising of the endothelial extension from both sides of the lumen and are about 2.5 μm in thickness (25-30 slices, Fig. 4C_1_–C_4_; Movie S4). Collagen fibers (Co) were also observed within the intussusceptive pillars and were oriented parallel to the endothelial circumference (Movie S5). Some of the endothelial extensions across the lumen were >2.5 μm thick, which depicted splitting or remodeling of the vessel (Fig. 5). In one region of interest, 4 pillars were observed spanning across the lumen and were plausibly involved in splitting of the CVP (Fig. 5B-B_3_; Movie S5). The 3D reconstruction of the scanned EM sections revealed distinct intraluminal connections of the intussusceptive pillars between the endothelial layers (Figs 4D and 5C and Movie S6). This evidence of tomographic reconstruction supporting the presence of intussusceptive pillars and their remodeling during CVP formation in zebrafish embryos is unprecedented. This interesting phenomenon of IA involved in maturation of vessels along with the clues of the RBC movement and increase in velocity prompted us to investigate more on hemodynamic profiles during CVP development.

### Simulation of blood flow dynamics during CVP development

**Figure 6:**
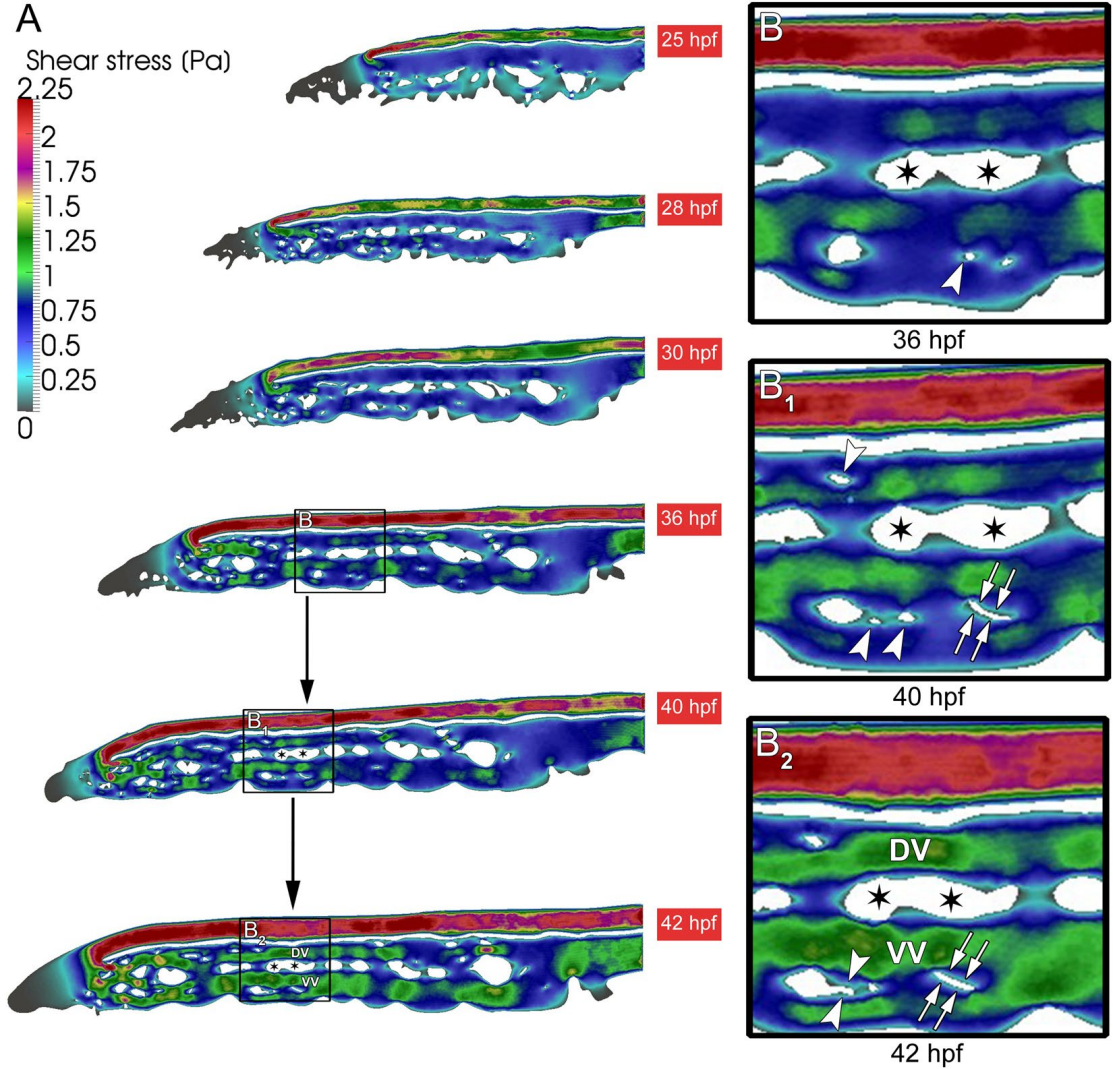
Shear stress distribution during CVP development. (**A**) The overall shear stress distribution calculated from blood flow videos obtained from *in vivo* microscopy between 25-42 hpf of developing CVP. (**B,B**_**1**_) Arrowheads show the appearance of pillars in the regions of declining shear stress. (**B**_**1**_,**B**_**2**_) Subsequently the same shear stress profile is associated with the path of pillar fusion indicated in white double arrows leading to the splitting of the vessel. The ensuing splitting of the vessels between 36-42 hpf leading to the formation of dorsal (DV) and ventral vein (VV). The splitting path is indicated with asterisks.

The correlation between mechanical forces and intussusceptive pillar formation during CVP development was studied by computational modeling (simulation). The in-house developed PakF software was used to simulate blood flow velocity and shear stress profiles in the dorsal aorta and CVP of *Tg(fli1a:eGFP)*^*y7*^ between 25-42 hpf. The velocity profile revealed that it remained high in the dorsal aorta (DA) corresponding to the color code in the scale bar (Fig. S1). During expansion of the CVP the velocity increased gradually by 30 hpf (300-400 µm/s) thereby increasing the area of perfusion and reached a peak by 42 hpf (400-500 µm/s) covering most of the regions especially during RBC drain back from the caudal artery to the CVP. Depending on the velocity gradient in the CVP, shear stress distributions were also evaluated (Fig. 6A). Shear stress increased in DA at 30 hpf from 1.5-2.0 Pa and reached a peak of 2.0-2.25 Pa by 42 hpf. The shear stress in the capillary network gradually increased from 0.25 Pa at 25 hpf to 1.25 Pa after the vascular network was established. Interestingly, we found that there was a steep drop in shear stress in the region of pillar formation and pillar fusion (Figs 6 and S2a). A sudden drop in shear stress of about 0.25-0.5 Pa was observed at 36 hpf (the arrowhead pointing the intussusceptive pillar region in Fig. 6B). During pillar fusion at 40 and 42 hpf (Fig. 6B_1_ and B_2_) and splitting, shear stress increased gradually between 1.0-1.25 Pa. Vascular splitting led to the formation of the Dorsal (DV) and Ventral (VV) vein and their shear stress profiles were plotted (Figs 6B_2_ and S2b). In addition, the change of value of shear stress over time at a specific point in CVP was plotted (Fig. S3a, b and c). In Fig. S3a pillar appearance was analyzed and it can be observed that the value of shear stress decreases when pillar appears. Pillar fusion is analyzed in Fig. S3b and it can be observed that the value of shear stress decreases when pillars are combined and then continues to increase over time. Finally, pillar expansion was analyzed in Fig. S3c and it can be observed that as the pillar grows, the value of shear stress also increases.

It should be noted that parameters defining characteristics of the blood in numerical simulations were approximated to be equal to the ones in humans. The main goal of blood flow simulations was to observe the changes in distribution of hemodynamic quantities, rather than just quantify them. These parameters only affect the values of the hemodynamic quantities, which was demonstrated in the sensitivity analysis. Additional simulations were performed to observe the effect of change of viscosity (Fig. S4) and inlet velocity (Fig. S5) on the maximum value of shear stress measured in the CVP at the time point 40 hpf. This proved that lower values of both parameters caused lower maximum shear stress and vice versa.

The data collected using numerical simulation raise the possibility that alteration within the blood flow and shear stress are essential for intussusceptive pillar formation and fusion, leading to vascular splitting. It seems that there is a specific shear stress constellation on the site of pillar formation (as indicated by blood flow velocity and shear stress (Fig. 6)). The computational flow dynamic simulation emphasizes the key factor for pillar formation and thus force driven remodeling of CVP.

### Intussusceptive angiogenesis is dependent on hemodynamics

Regulation of hemodynamic parameters, like blood velocity and shear stress seems to affect IA. Pharmacological compounds like adrenergic receptor agonist isoprenaline hydrochloride (isoprenaline) and 2,3-butanedione monoxime (BDM, a myosin ATPase inhibitor) were used to increase or decrease the heart rate, respectively. These treatments mimic physiological and pathological blood flow conditions that can occur during formation of the CVP in the zebrafish embryo.

**Figure 7:**
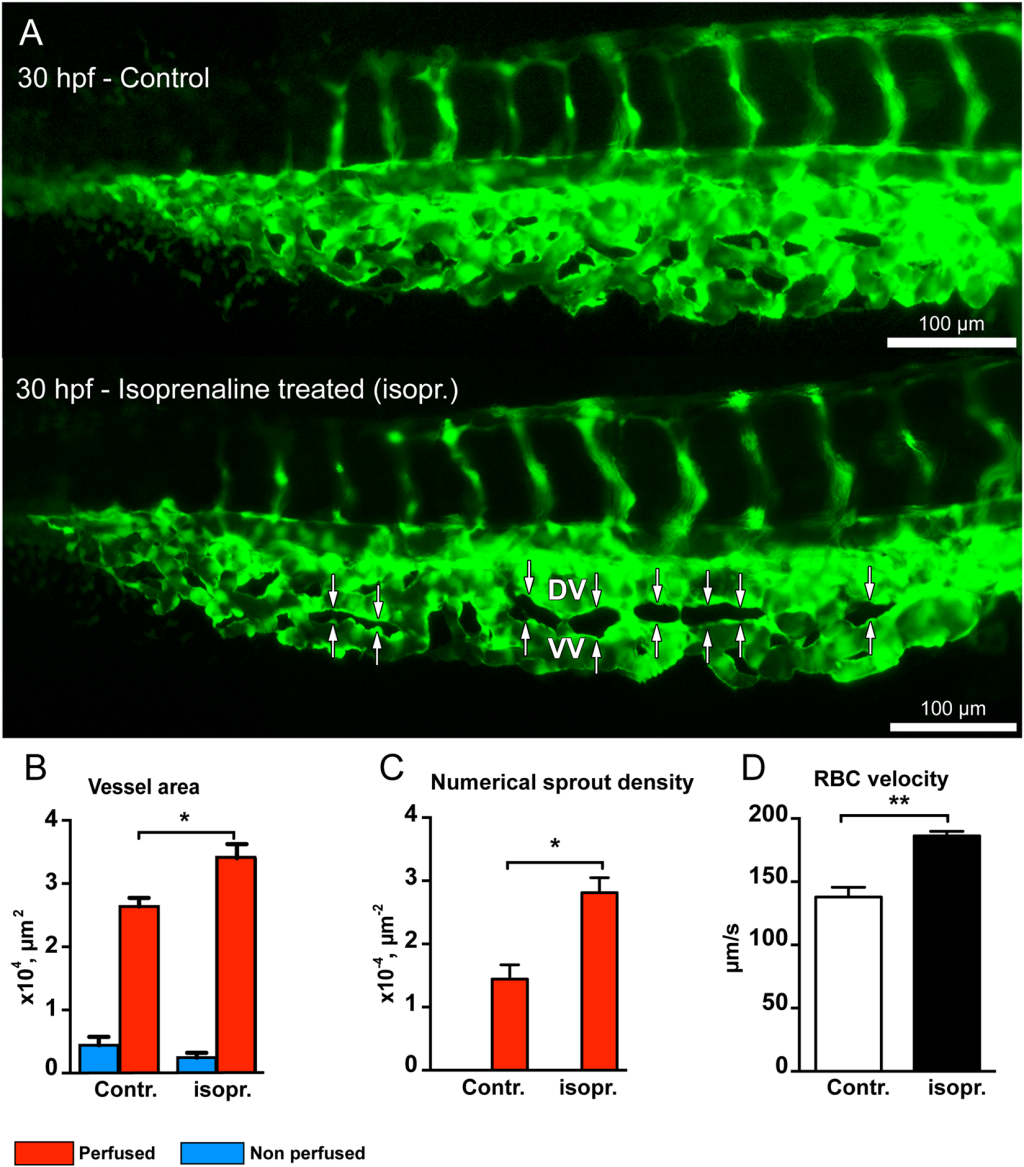
Isoprenaline hydrochloride (Isopr.) treatment accelerates vascular development and splitting in the zebrafish CVP. (**A**) CVP of the control embryo at 30 hpf compared with that of isoprenaline-treated embryo. The white double arrows marked in the treated CVP represent advanced vascular splitting and segregation of the DV and VV in comparison to the control CVP. (**B**) The treated CVP resulted in significant increase in vessel area and (**C**) increased numerical pillar density in the perfused region. (**D**) The treated embryos showed significant increase in RBC velocity compared to non-treated zebrafish embryos.

**Figure 8:**
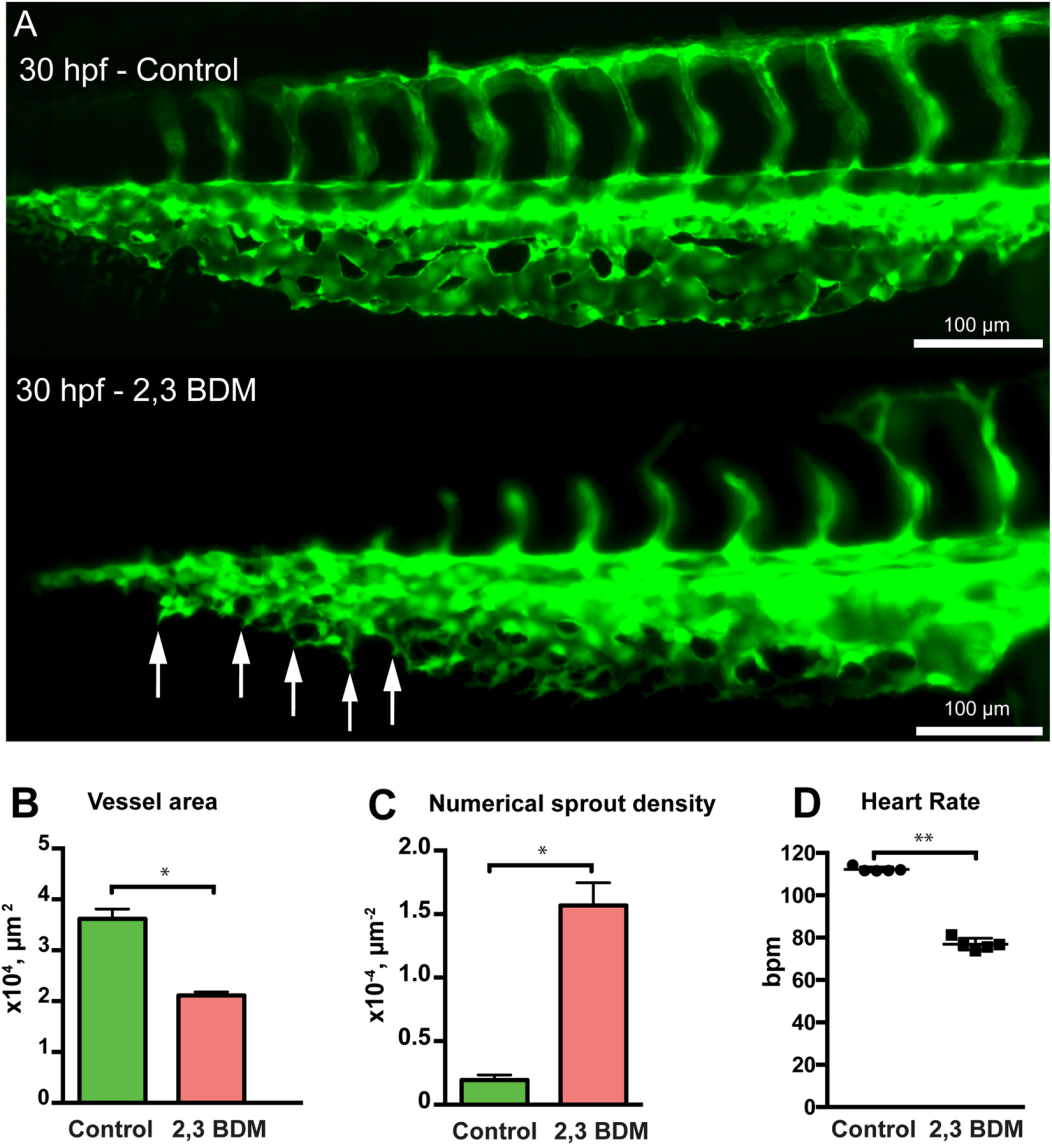
Treatment with 2,3 BDM decreases CVP development in zebrafish embryos. (**A**) The CVP of the control compared with 2,3 BDM treated embryos at 30 hpf. The treated CVP shows numerous sprouts (white arrows) and delay in growth at 30 hpf in comparison with the control. (**B**) The 2,3 BDM treated CVP shows significant decrease in vessel area. (**C**) The numerical sprout density has increased immensely in the treated embryos. (**D**) The heart rate of the 2,3 BDM treated embryos shows significant decrease compared to control embryos at 30 hpf.

At 30 hpf, the RBC velocity in the CVP was increased significantly (30%) in isoprenaline treated embryos (186.3 ± 3.5 µm/s) compared to non-treated embryos (137.9 ± 7.8 µm/s, Fig. 7D). This increase in blood flow in the CVP resulted in significant acceleration in growth of the isoprenaline treated CVP. The isoprenaline treated CVPs at 30 hpf showed dramatically enhanced growth (Fig. 7A) and the morphological evaluation of the vessel area of isoprenaline treated embryos showed remarkable increase (28%) compared with the vessel area of the non-treated CVP (Fig. 7B). Interestingly, the perfused region of the isoprenaline treated CVP showed marked increase in the numerical pillar density (44 ± 4%, Fig. 7C) in contrast to the perfused region of the control CVP. This resulted in initiation of splitting of CVP into DV and VV at an early phase of embryonic development (30 hpf) due to the increase in blood flow with isoprenaline treatment (Fig. 7A). On the contrary, BDM treatment reduced blood flow in the CVP with decrease (32%) in the mean heart rate to 76 ± 1.2 beats per minute (bpm) in comparison to 112.3 ± 0.5 bpm recorded in non-treated embryos at 30 hpf (Fig. 8D). This reduction in the vessel perfusion distinctly diminished the vessel area of the CVP from 3.6 ± 0.9 × 10^4^ µm^−2^ in control to 2.1 ± 0.3 × 10^4^ µm^−2^ in BDM treated embryos (Fig. 8B). Absence of perfusion in the BDM treated CVP resulted in absolute lack of pillar formation; however, numerous angiogenic sprouts dominated the entire CVP (Fig. 8A). The numerical sprout density for BDM treated CVP was at 15.0 ± 0.8 × 10^−4^ µm^−2^, this density was found to be significantly higher than the sprout density of 1.9 ± 0.2 × 10^−4^ µm^−2^ in the control CVP (Fig. 8C). Drastic phenotypic changes in CVP formation were observed in the BDM treated embryos. The CVP was retained in the active sprouting phase and lacked vascular integrity, progression towards intussusceptive phase and remodeling due to the absence of blood flow. However, the obtained results show that the flow dynamics plays an important role but these treatments affect more than the blood flow velocity while it cannot be used to infer the direct effect of low/high shear stress. Our results strongly suggested that blood flow modulation critically influenced intussusceptive pillar formation and IA.

In parallel to pharmacological modulation of hemodynamics, morpholino antisense oligonucleotides of *troponin 2* (*tnnt-2*), which inhibits cardiac output and a *gridlock (grl)*, specifically inhibits blood flow in the trunk and the tail of zebrafish embryo (Fig. S6a and b). Due to lack of blood flow, development of the CVP is impaired in both morphants. It is obvious that the number of pillars is dramatically reduced in the morphants. The absence of blood flow completely inhibited remodeling of the CVP and thereby impaired the segregation of DA and CVP. These data evidently support the fact that blood flow is essential for IA mediated remodeling of CVP.

## Discussion

Developing capillary plexuses have to expand rapidly and satisfy the oxygen and nutrient demand of the growing embryo. At the same time the latter should adapt to the changing hemodynamic conditions i.e. increased blood pressure and blood velocity. The augmentation of the vasculature is achieved by angiogenesis, while vascular remodeling and pruning are responsible for hemodynamic optimization and hierarchical organization of the embryonic vasculature.

Following the initial observation of IA in vascular casts of rat pulmonary vessels in the late 80s, the process between SA and IA has been documented only morphologically. It has been demonstrated in the chicken developing CAM, kidney and lung that after the initial formation of the capillary plexus by SA additional augmentation and remodeling are achieved by intussusception^12,13^. It seems that IA could be “switched on” after initiation of anti-VEGF therapy in tumors^29^. The exact mechanisms of action between SA and IA were never documented *in vivo*. Zebrafish embryos have proven to be an excellent model for investigating SA^30^, but the existence of IA during zebrafish embryonic development has remained hypothetical until a recent study that has documented structures resembling intussusceptive pillars during the development of CVP^31^.

We asked if the intussusceptive angiogenesis mechanism does occur during zebrafish vascular development and started to investigate in *Tg(fli1a:eGFP)*^*y7*^ zebrafish embryos. The developing CVP from 24 to 42 hpf was captured using *in vivo* fluorescence imaging, similar to previous findings^32^. Numerous outgrowths of angiogenic sprouts, which initiate CVP growth at 24 hpf mostly in the non-perfused regions were observed. These sprouts interconnect each other to form the primordial plexus by 28 hpf. In the chick embryo CAM model, intussusceptive microvascular growth was shown to be mainly responsible for the capillary network expansion following an earlier sprouting phase^1,6^. Similarly, during embryonic development of the CVP in the zebrafish embryos, increase in the area of perfusion at 36 hpf leads to the appearance of tiny intussusceptive pillars, which fuse with the neighboring ones and result in remodeling of the CVP.

Three-dimensional visualization of intraluminal protrusions of intussusceptive pillars is essential for the characterization of IA^11^. The 3D reconstruction of transluminal intussusceptive pillars using *in vivo* confocal laser scanning microscopy and SBF-SEM further confirmed the existence of IA in the developing zebrafish CVP. The 3D finite element models helps to study intussusceptive angiogenesis on various models depending upon the flow dynamics^25^. Future studies on the IA should focus on dynamics of pillar formation and the concomitant cellular events including loss of endothelial cell polarity, rearrangement of the cytoskeleton, creation of endothelial-endothelial junctions (so called kissing contacts = first step of pillar formation). Plausibly, such investigations would reveal new anti-angiogenic targets applicable in anti-angiogenesis based therapy.

During zebrafish CVP development, the angiogenic sprouts are initially not perfused by blood whereas the intussusceptive pillars are formed in the perfused vascular areas. Blood flow and shear stress have a major role in segregation of straight capillary segments smaller feeding and draining microvessels; a process termed before intussusceptive arborization^6^. The described specific flow profile with a drop in shear stress resulted in following events running simultaneously (1) “expansion, reshaping and fusion of already existing serried pillars” (how they arose is secondary; this could be by vasculogenesis, fusion of sprouts or intussusception); (2) “formation and subsequent fusion of the new pillars with the serried elongated pillars and slits as described in “1”. Repetitive formation and fusion of such kind of pillars resulted in delineation of straight capillary segments and generation of smaller future feeding and draining microvessels. The concept of pruning was introduced by Ashton investigating development of retinal vessels under hypoxia which refers to the process of “removal of superficial branches”^33^. It is clear that it is conceptually incorrect to name the formation of the new vascular entities described as “pruning” because it reduces the number of vascular segments. The role of the intussusceptive pillar formation in the real pruning of superficial branches has been described previously in different models^29,34,35^. Due to the fact, that the newly arrived pillars are transient structures (they fuse in a short time with the serried elongated pillars and slits) and on the end the vascular complexity decreases. This does not justify naming the process as “angiogenesis”. Therefore, it will be reasonable to stick to the previously introduced term“intussusceptive arborization”^6,35^. The latter described exactly the series of events that we observed in CVP.

Several studies have shown in different animals models such as mouse, rat and zebrafish that a link exists between hemodynamics and vascular remodeling, regression and pruning^36–38^. In the recently published review by Richard and Simons^39^ the authors evaluated different mechanisms of vascular pruning. Lenard *et al*. reported cellular mechanisms involved in the process; type I pruning involves apoptosis, including endothelial cell apoptosis and type II pruning was apoptosis independent. The latter type entails retraction and migration of endothelial cells^40^. The second type of pruning mainly depends upon the presence of blood flow where the endothelial cells rearrange accordingly, collapse and undergo self-fusion to separate the lumen^40^. In the present study, the existence of intussusceptive pillar formation was documented along with vascular regression and intussusceptive arborization. The intussusceptive arborization observed in the CVP during zebrafish embryonic development is similar to type II cellular pruning described by Lenard *et al*.^40^.

The 3D computational flow simulations of the chick CAM have shown a significant role of wall shear stress and specifically, the areas of a dramatic drop in shear stress are known to induce pillar formation and remodeling in the developing chick CAM vessels^7^. In our study, shear stress distribution was also mapped between 25 hpf to 42 hpf using RBC velocities in the perfused regions of the CVP. The computational model of CVP confirmed our previous findings^8,41^ that the transluminal intussusceptive pillars are formed in regions with drastic drop in shear stress within a short distance. With the increasing blood velocity during development, the areas with shear stress discrepancy become more pronounced, what induces progressive pillar fusion and splitting thus leading to intussusceptive arborization. This alteration to shear stress in the vessels correlates with the structural changes in the microcirculatory network mainly due to transluminal pillar formation^25,42^. Experimental changes to shear stress by accelerating blood flow in chick CAM vessels has proven the association between blood flow dynamics and pillar formation^8^.

In agreement with previous reports in zebrafish embryos^43,44^ by using isoprenaline hydrochloride and 2,3 BDM, similar rates of increase or decrease in the heart rate respectively were observed. To evaluate the role of circulatory flow during morphogenesis of the CVP, isoprenaline hydrochloride treatment accelerated the growth of the CVP. This was mainly by intussusceptive arborization and early remodeling of the CVP was inferred. In contrast, reducing the heart rate using the 2,3 BDM resulted in decreased growth of the CVP, which was dominated by the sprouting phase throughout the drug treatment period and lacked intussusceptive arborization possibly due to absence of blood flow. Our results were congruent with vessel remodeling studies on zebrafish cranial division of internal carotid artery during eye blood vessel development^45^. Blocking the heartbeat pharmacologically or by genetic manipulation induced blood vessel pruning whereas re-stimulation prevented vessel pruning^36^. The blood flow dependency of intussusceptive arborization and vascular regression and intussusceptive pruning has motivated further investigations on mechanical forces^39^. From our analyses and according to the literature, we understood that intussusceptive arborization and vascular regression are driven by hemodynamics and play an essential role in the formation of hierarchical vascular tree

In addition, to overcome the possible nonspecific phenotype due to side effects of pharmacological modulators, a more precise genetic modification by anti-sense oligonucleotides known to inhibit translation of proteins by injecting morpholino in zebrafish embryos was used^46^. The lack of circulation in tnnt-2 morphants severely impaired growth of both ISVs and CVP. No intussusceptive pillar formation, vascular regression and intussusceptive arborization has been observed but numerous angiogenic sprouts drove the growth of the impaired CVP due to lack of perfusion. Interestingly, sequestering blood flow to the fin vessels by aortic coarctation in *grl* mutants has shown near normal development of ISVs and CVP. Anastomoses of the numerous sprouts appearing in the non-perfused areas mainly drives growth of the CVP, nevertheless the *grl* morphants lacked pillar formation and remodeling for the segregation of caudal veins into DV and VV. The one possible explanation for the near normal development of the CVP in the *grl* morphants is that, in these mutants with functional hearts, there develops collateral vessels and they survive to adulthood^47,48^. We infer from our results that hemodynamics regulate IA by pillar formation and are also involved in post angiogenic mechanisms, namely blood vessel regression and intussusceptive pruning^38,39^.

In conclusion, we have studied the role of intussusceptive arborization in the zebrafish CVP model (*in vivo*) with the observation that sprouting initiates the primitive CVP and the expansion of the capillary plexus is driven by the intussusceptive arborization. This process occurs in perfused regions showing decline in shear stress confirming that intussusceptive arborization is mainly regulated by hemodynamics. Thus, the synergistic actions between the sprouting and intussusception can be evaluated simultaneously within few hours during the morphogenesis of CVP model in zebrafish embryos.

## Electronic supplementary material

Supplementary Figures

Supplementary video S1

Supplementary video S2

Supplementary video S3

Supplementary video S4

Supplementary video S5

Supplementary video S6
