# Publisher Correction: Synergistic interaction of sprouting and intussusceptive angiogenesis during zebrafish caudal vein plexus development

**DOI:** 10.1038/s41598-018-36663-y

**Published:** 2019-03-06

**Authors:** Swapna Karthik, Tijana Djukic, Jun-Dae Kim, Benoît Zuber, Andrew Makanya, Adolfo Odriozola, Ruslan Hlushchuk, Nenad Filipovic, Suk Won Jin, Valentin Djonov

**Affiliations:** 1 Institute of Anatomy, University of Bern, Bern, Switzerland; 2 Graduate School for Cellular and Biomedical Sciences, University of Bern, Bern, Switzerland; 3 Research and Development Center for Bioengineering, BioIRC, Sretenjskogustava 27, Kragujevac, Serbia; 4 Faculty of Mechanical Engineering, University of Kragujevac, SestreJanjic 6, 34000 Kragujevac, Serbia; 5 Yale Cardiovascular Research Center and Section of Cardiovascular Medicine, Department of Internal Medicine, Yale University School of Medicine, New Haven, CT 06511 USA; 6 School of Life Sciences and Cell Logistics Research Center, Gwangju Institute of Science and Technology, Gwangju, Korea; 7 Department of Veterinary Anatomy and Physiology, University of Nairobi, Nairobi, Kenya

Correction to: *Scientific Reports* 10.1038/s41598-018-27791-6, published online 29 June 2018

In the Supplementary material originally published with this Article, Supplementary Videos 4 and 5 did not display correctly. This has been corrected in the HTML version of the Article; the PDF version was correct at the time of publication.

