# Supplementary material for: Synergistic interaction of sprouting and intussusceptive angiogenesis during zebrafish caudal vein plexus development
Source: Sci Rep. 2018 Jun 29;8:9840. doi: 10.1038/s41598-018-27791-6 (PMC6026200; doi:10.1038/s41598-018-27791-6)
Supplement: Supplementary file 1 — Supplementary Figures [file 41598_2018_27791_MOESM1_ESM.pdf]

Synergistic interaction of sprouting and intussusceptive angiogenesis during zebrafish caudal vein plexus development

Swapna karthik[1,2],Tijana Djukic[3,4],Jun-Dae Kim[5],Benoît Zuber[1],Andrew Makanya[1,7], Adolfo Odriozola[1],Ruslan Hlushchuk[1],Nenad Filipovic[3,4],Suk Won Jin[5,6] and Valentin Djonov*[1]

Affiliations:

[1]Institute of Anatomy, University of Bern, Bern, Switzerland.

[2]Graduate School for Cellular and Biomedical Sciences, University of Bern, Switzerland.

[3]Research and Development Center for Bioengineering, BioIRC, Sretenjskog ustava 27, Kragujevac, Serbia.

[4]Faculty of Mechanical Engineering, University of Kragujevac, Sestre Janjic 6, 34000, Kragujevac, Serbia.

[5]Yale Cardiovascular Research Center and Section of Cardiovascular Medicine, Dept. of Internal Medicine, Yale University School of Medicine, New Haven, CT, 06511, USA.

[6]School of Life Sciences and Cell Logistics Research Center, Gwangju Institute of Science and Technology, Gwangju, Korea.

[7]Department of Veterinary Anatomy and Physiology, University of Nairobi, Nairobi, Kenya.

Please address correspondence to:
Prof. Valentin Djonov, MD
Professor and Chair
Institute of Anatomy
University of Bern
Baltzerstrasse 2
CH-3000 Berne-9 Switzerland
Phone: +41 31 631 8432 / 8431 Fax: +41 31 631 3807
e-mail: valentin.djonov@ana.unibe.ch

Supplementary figures:

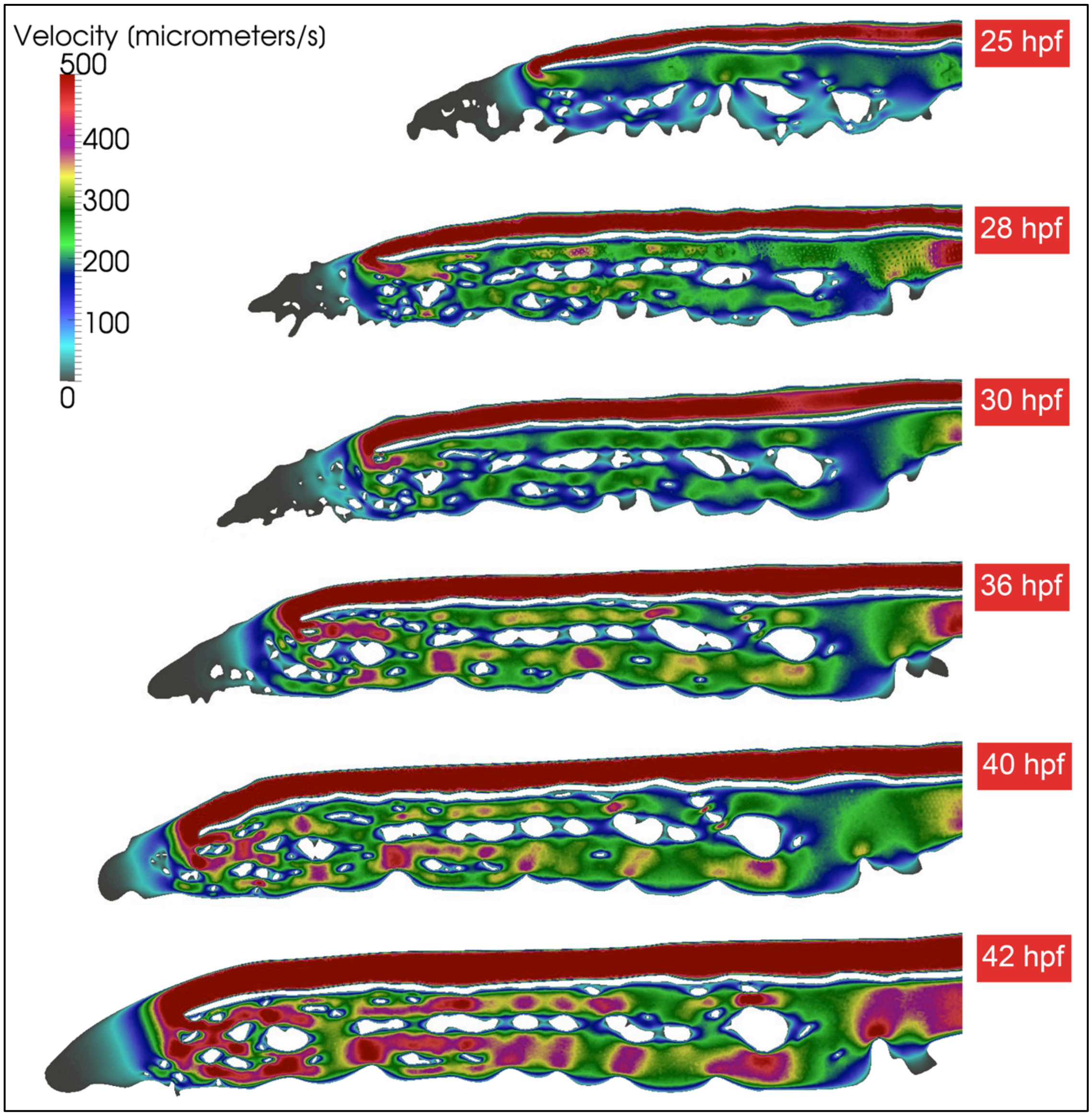

Fig.S1: Overall RBC velocity distribution in the developing CVP of zebrafish embryo.

RBC velocity was measured in the dorsal aorta and in the CVP in µm/s between 25 hpf-42 hpf. The velocity increased with time during formation of the CVP, leading to the increase in area of perfusion and thus intussusception in the perfused region.

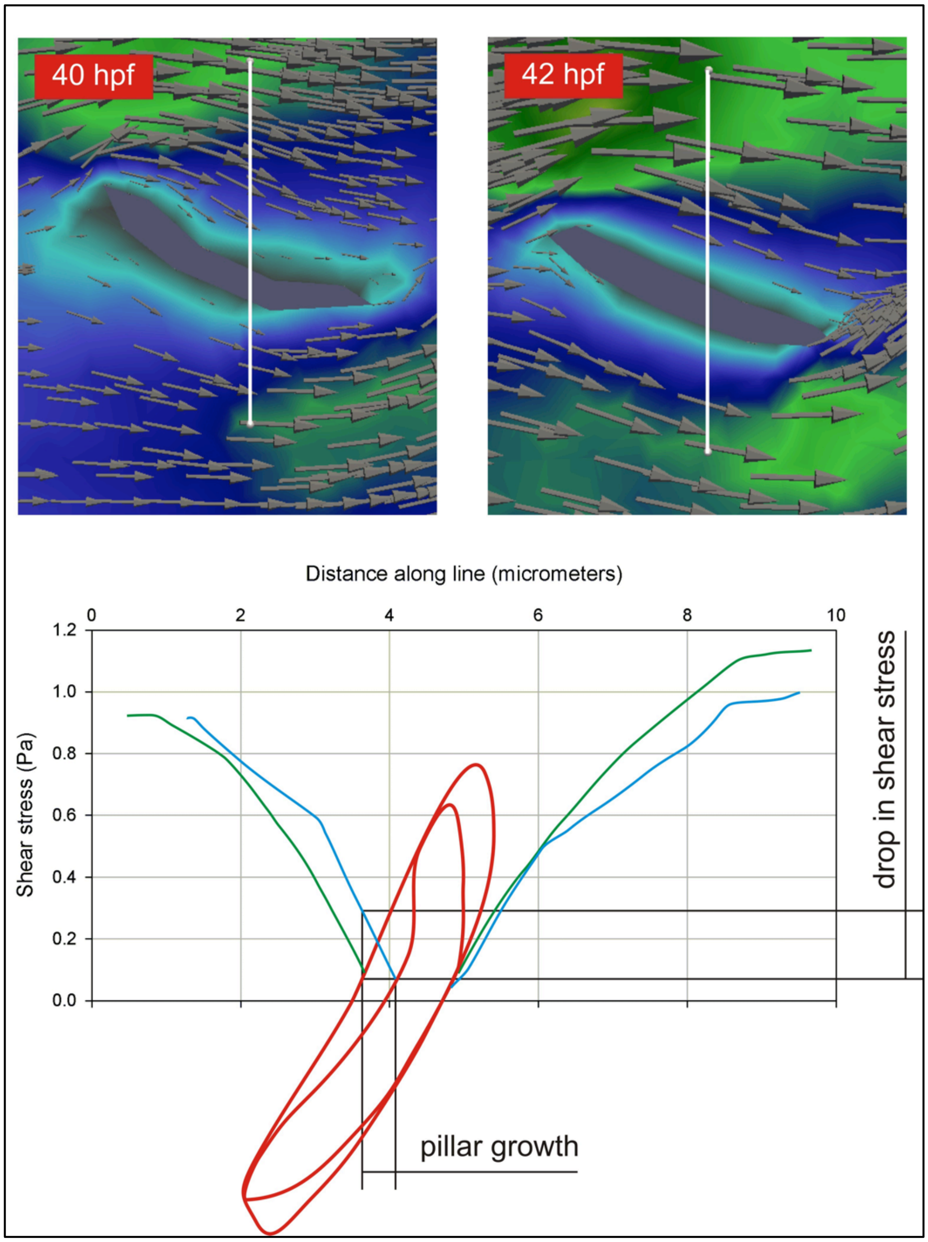

Fig.S2a: Shear stress variation with respect to distance from the pillar in the intussusceptive pillar region.

The figures above the graph are enlarged images of the pillar analyzed (indicated by single arrows in Fig. 4) at 40 and 42 hpf, with arrows indicating the direction of shear stress vectors. White lines on these images denote the direction along which the shear stress values are plotted in the graph. The blue line in the graph represents the shear stress variation at 40 hpf, while the green one indicates shear stress variation at 42 hpf. The pillar shape is illustrated with red lines. As can be seen from the graph, the drop in shear stress can be correlated to the pillar growth between the two time points.

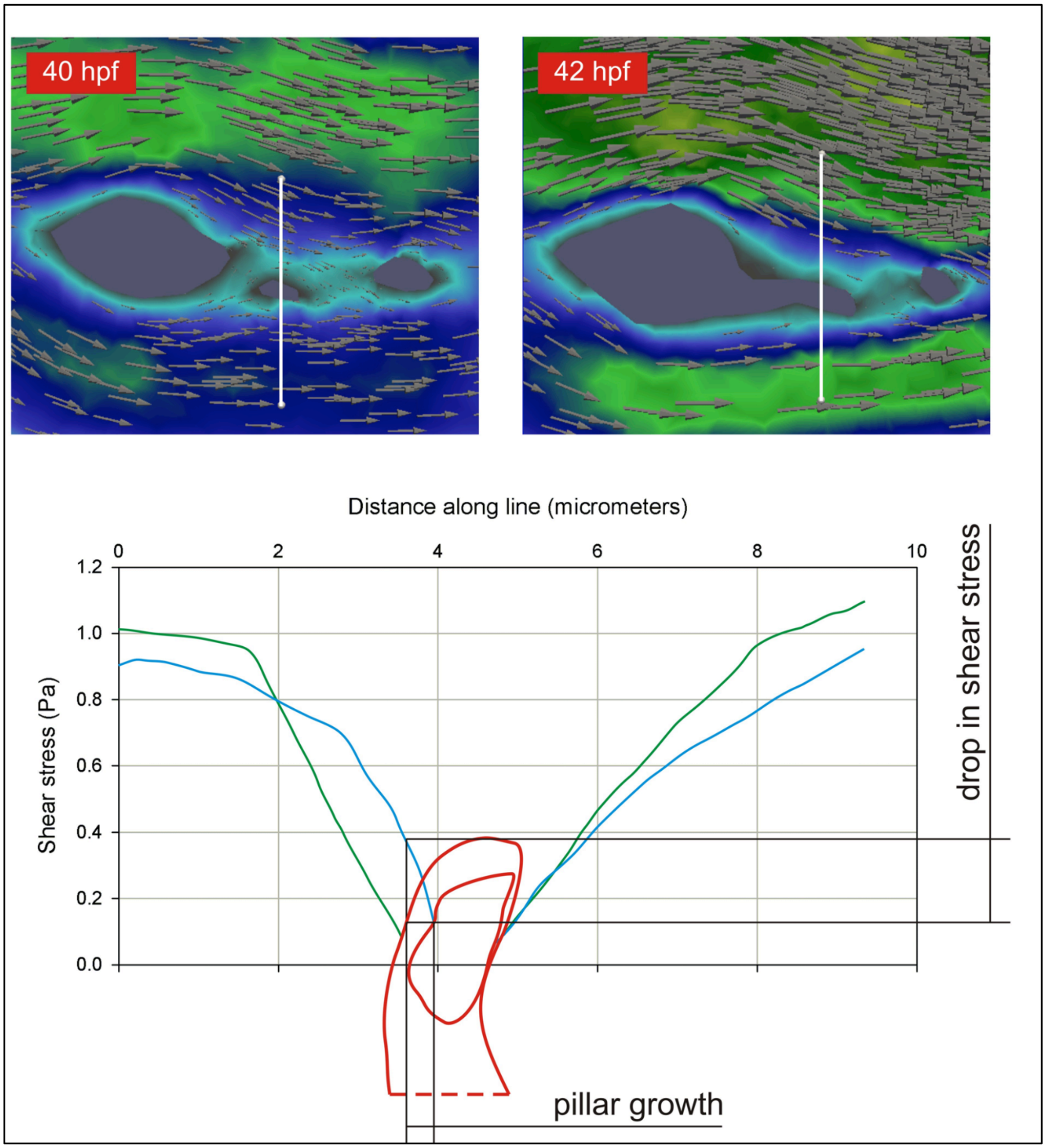

Fig.S2b: Shear stress variation with respect to distance from the pillar during pillar fusion.

The images above the graph are enlarged representations of the analyzed pillar (indicated by double arrows in Fig. 4) at 40 and 42 hpf, with arrows indicating the direction of shear stress vectors. White lines on these images denote the direction along which the shear stress values are plotted in the graph. The blue line in the graph represents the shear stress variation at 40 hpf, while the green one indicates shear stress variation at 42 hpf. The pillar shape is illustrated with red lines.

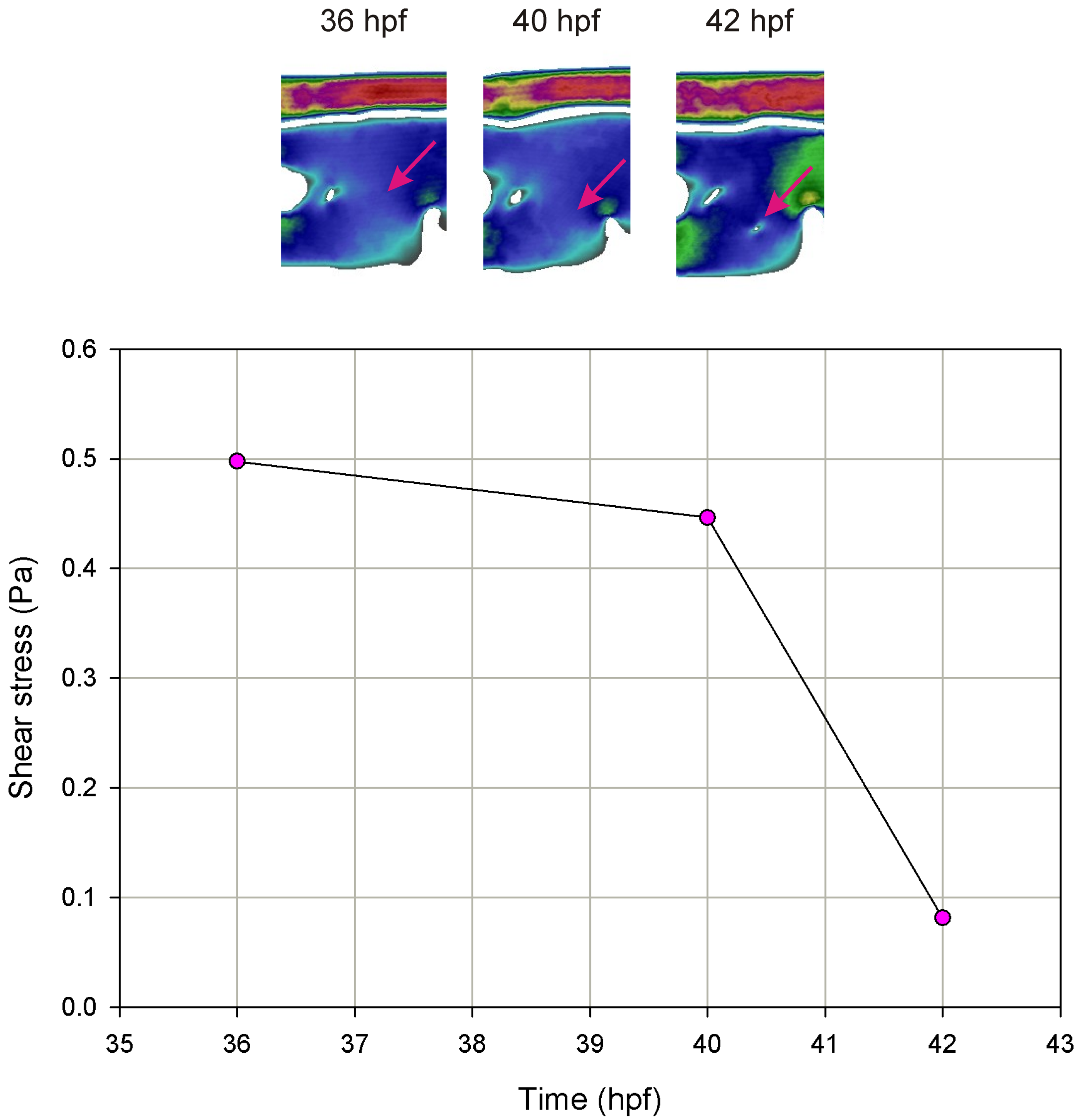

Fig.S3a: Change of shear stress with respect to time, for a particular position in the CVP, illustrating pillar appearance.
Above the graph are the segments of the CVP, with pink arrows indicating the observed position.

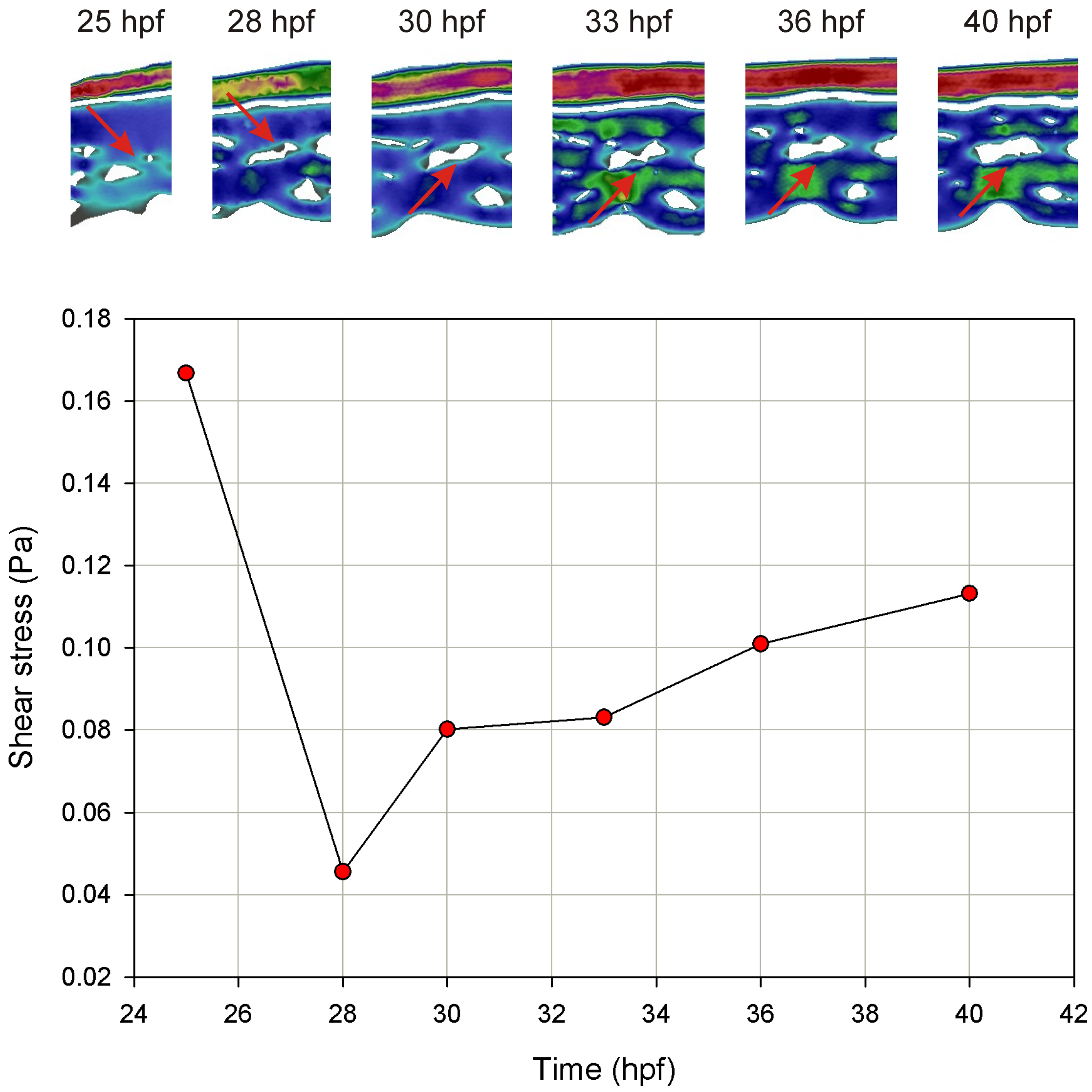

Fig.S3b: Change of shear stress with respect to time, for a particular position in the CVP, illustrating pillar fusion.
Above the graph are the segments of the CVP, with red arrows indicating the observed position.

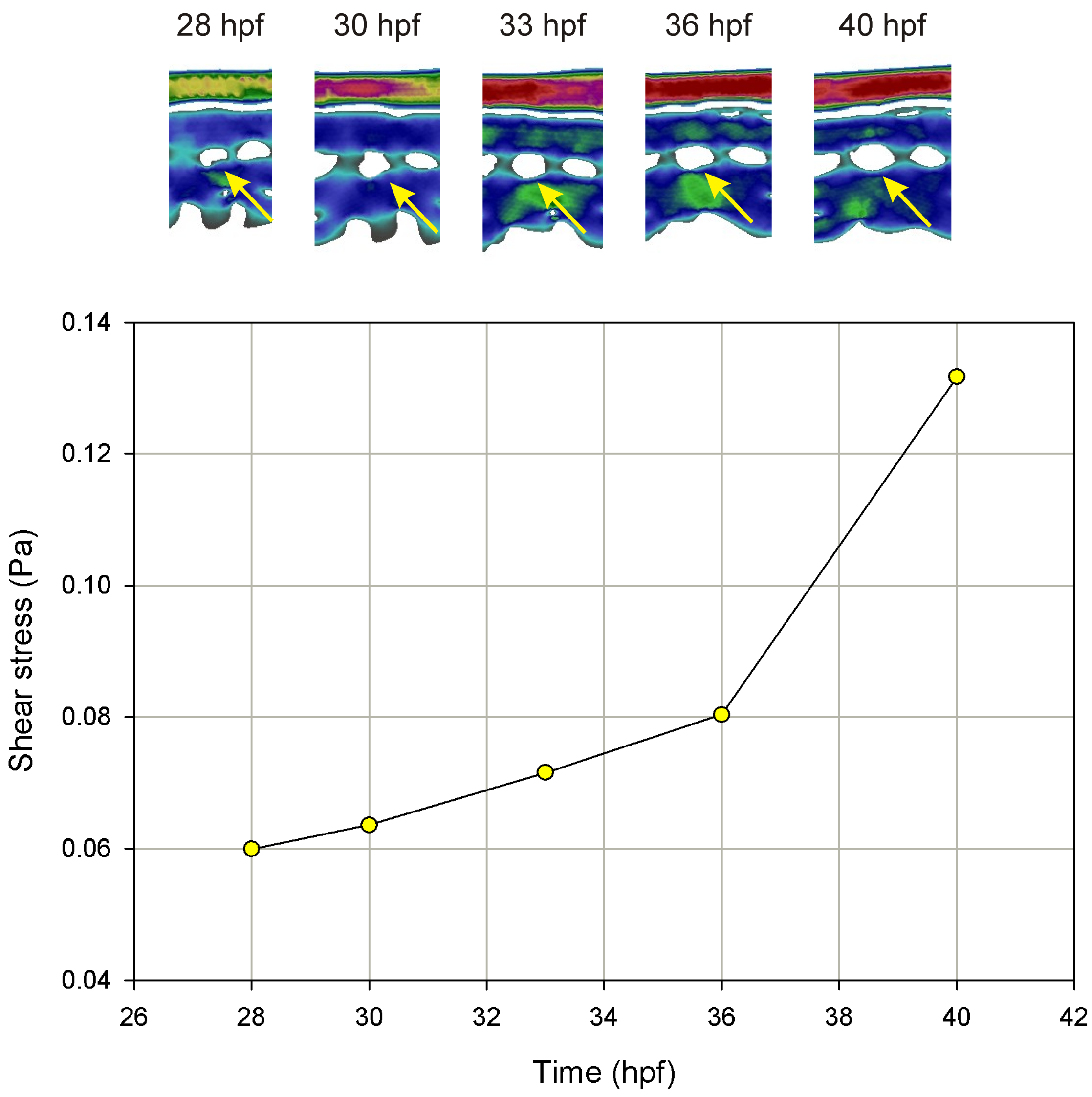

Fig.S3c: Change of shear stress with respect to time, for a particular position in the CVP, illustrating pillar expansion.
Above the graph are the segments of the CVP, with yellow arrows indicating the observed position.

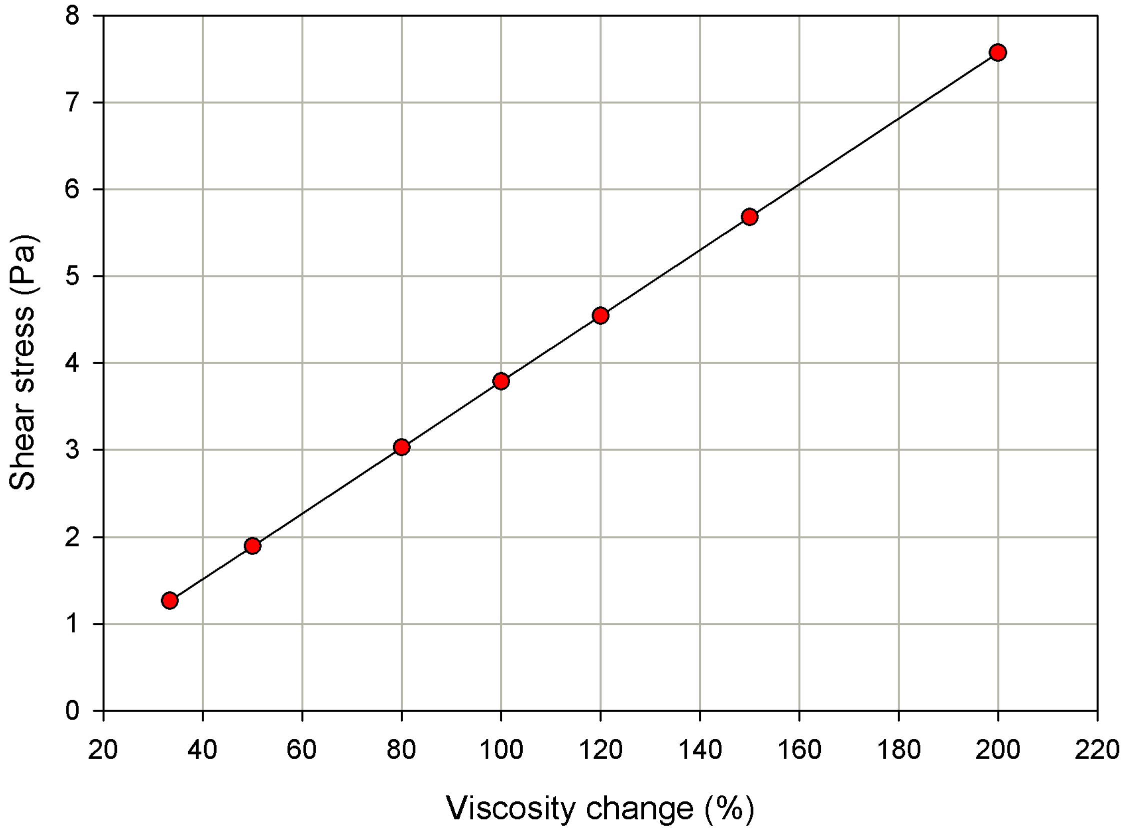

Fig.S4: Results of the sensitivity analysis.
Parameter of the numerical model defining viscosity of the blood was varied and the influence of this change on the maximum value of shear stress measured in the CVP, 40 hpf was analyzed.

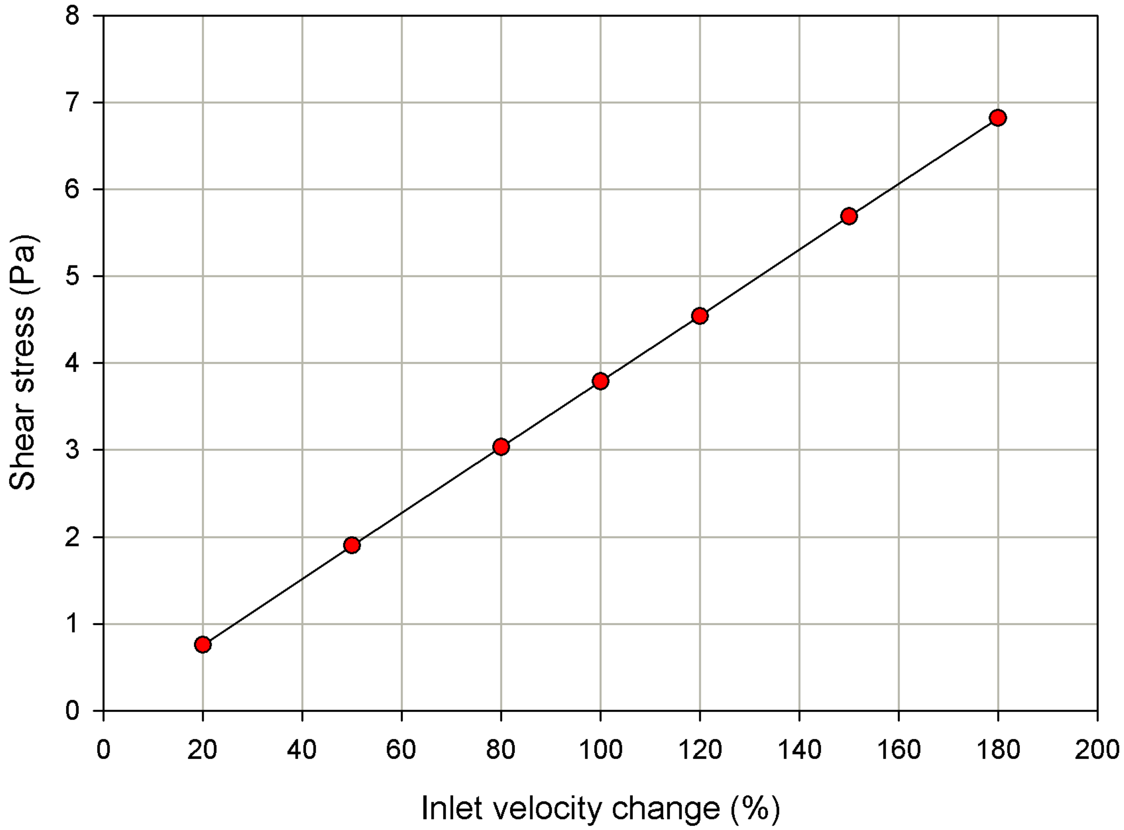

Fig.S5: Results of the sensitivity analysis.
Parameter of the numerical model defining velocity prescribed at the inlet was varied and the influence of this change on the maximum value of shear stress measured in the CVP, 40 hpf was analyzed.

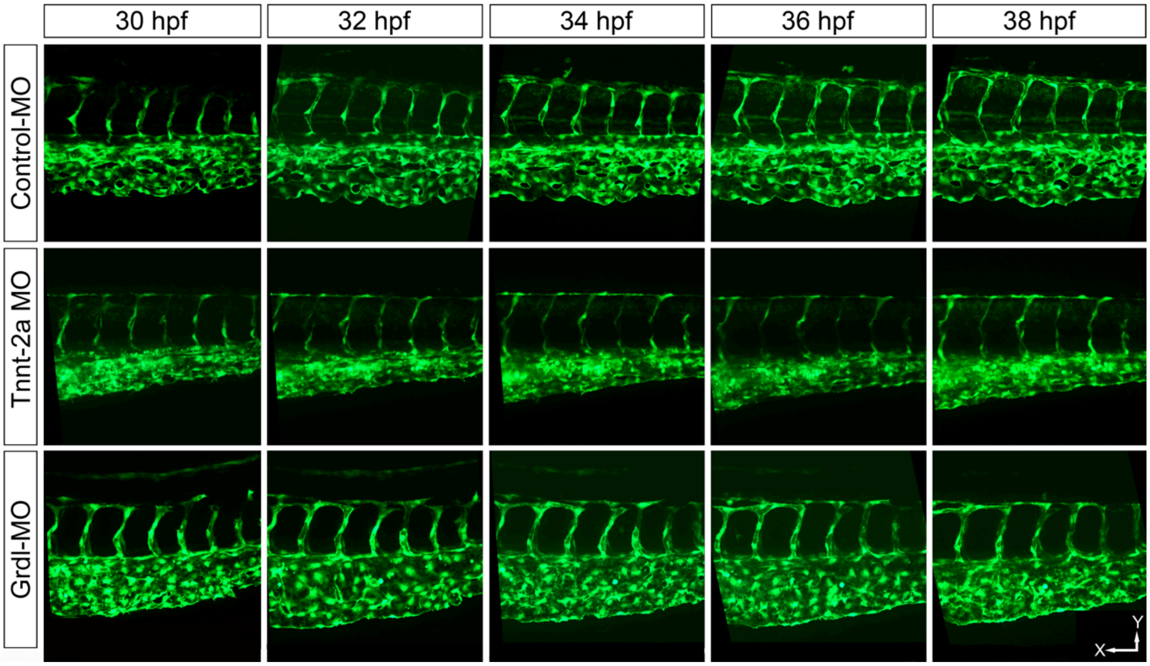

Fig.S6a: Morpholino (MO) antisense oligonucleotides microinjected CVP between 30-38 hpf.

The control MO injected CVP shows normal CVP development, which remodels with time. While tnnt-2a and gridlock MO injected embryos show impaired CVP development along with the dramatically reduction in pillar formation. Scale bar x-axis = 50 μm; y-axis=32 μm. See also supplementary figure S3.

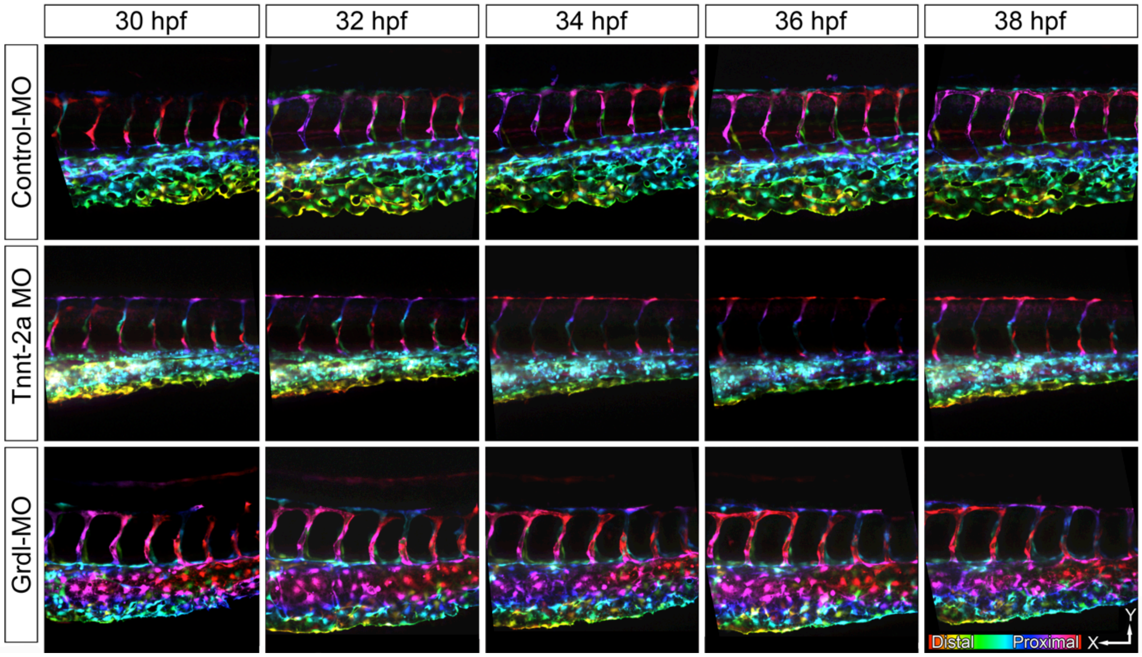

Fig.S6b: Morpholino (MO) antisense oligonucleotides microinjected CVP between 30-38 hpf.
Z-stacks of the CVP were depth image-coded according to their z positions. The color code bar represents the scanning from proximal to distal regions of the CVP.

Supplementary Materials and Methods:

Computational simulation and software details

The blood flow is considered to be three-dimensional and blood is considered as a viscous incompressible fluid. The flow is governed by the Navier-Stokes equations and continuity equation that can be written as:

$$\rho\left(\frac{\partial v_i}{\partial t}+v_j\frac{\partial v_i}{\partial x_j}\right)=-\frac{\partial p}{\partial x_i}+\mu\left(\frac{\partial^2 v_i}{\partial x_j \partial x_j}+\frac{\partial^2 v_j}{\partial x_j \partial x_i}\right) \quad (1)$$

$$\frac{\partial v_i}{\partial x_i}=0 \quad (2)$$

where v_i is the blood velocity in direction x_i, ρ is the fluid density, p is pressure, μ is the dynamic viscosity; and summation is assumed on the repeated (dummy) indices, i,j=1,2,3. The first equation represents balance of linear momentum, while the second equation represents the incompressibility condition.

The wall shear stress is calculated as

$$\tau=-\mu\frac{\partial \vec{v}_t}{\partial \vec{n}} \quad (3)$$

where $\vec{v}_t$ denotes the tangential velocity close to the walls, and $\vec{n}$ is the normal direction at the vessel wall. The velocity field is obtained by solving equations (1) and (2). Tangential velocity near the wall surface is used to numerically evaluate the velocity gradient $\frac{\partial \vec{v}_t}{\partial \vec{n}}$. This way, 3 components (τ_x,τ_y,τ_z) of the wall shear stress vector $\vec{\tau}$ are obtained. The effective value of wall shear stress τ_{eff} is calculated as:

$$\tau_{eff}=\sqrt{\tau_x^2+\tau_y^2+\tau_z^2} \quad (4)$$

The boundary conditions for the simulation are set such that the velocity at the walls of the domain is equal to zero. The velocity at the inlet of the domain is prescribed and that represents the initial condition. The value of velocity that is prescribed at the inlet is calculated using experimental data, by tracking several individual red blood cells using imaging techniques. The obtained values of velocity for individual RBCs are averaged and used for the inlet velocity, to ensure higher accuracy. At the outlet, the outflow zero pressure boundary condition is applied. In the simulations, the convergence criterion is defined such that the maximum absolute change of velocity between two respective time steps must be less than 10^{-3}.

A finite element mesh with 3D 8-node finite elements was generated for all the models. The number of elements in the meshes used in numerical simulations in this study was from 340000 to 530000. Mesh independence study was performed, to ensure the accuracy of results. This study was performed such that first an initial mesh was created with around 150000 elements.Then the mesh was refined in an iteration loop, until the results were no longer changing more that the defined tolerance. More precisely, until the change of shear stress values was less than 2%. Overall 7 different mesh resolutions were generated and the mesh indenpence was reached at 387.000 finite elements, for the control case. All other models were then generated accordingly.

In numerical simulations the units for physical dimensions of the domain are micrometers, obtained velocity distribution is shown in micrometers/second, obtained pressure and wall shear stress distributions are shown in Pascal (N/m^2). Characteristics of blood are defined as follows: density is set to 1.05 g/cm^3, dynamic viscosity is set to $3.675 \cdot 10^{-3} \mathrm{Pa} \cdot \mathrm{s}$. These parameters were chosen according to the available data for humans. This approximation can however be justified by the fact that in this study the main goal was to observe the distribution and relative change of quantities related to blood flow, rather than simply quantify them.
Additional sensitivity analysis is performed, to observe the effect of change of viscosity (Fig. S4) and inlet velocity (Fig. S5) on the maximum value of shear stress measured in the CVP in the time point 40 hpf. As it was expected, the lower values of both parameters cause lower maximum shear stress and vice versa.

Supplementary movie legends:

Supplementary movie S1
Overview of blood flow in the developing CVP showing the increase in perfusion between 24 - 42 hpf.

Supplementary movie S2
In vivo observation of the perfused region prior to and after pillar formation incorporating also the time of pillar fusion and splitting between 34–40 hpf.

Supplementary movie S3:
3D reconstruction of intussusceptive pillar of 38 hpf - reconstructed from the z stack projections of laser scanning microscope. The movie was created using Imaris software.

Supplementary movie S4
Serial block face electron microscopy of CVP (3 view) of 38 hpf zebrafish embryo showing a developing CVP. Appearance of a pillar in in the CVP in the ventral region of the embryo is indicated with an arrow. The other subcellular structures such as erythrocytes, endothelial cells and inter-endothelial junctions were observed. The movie was created from the stack of sections using Fiji software.

Supplementary movie S5
Serial block face electron microscopy of CVP showing the appearance of 4 intussusceptive pillars spanning across the lumen (about 500 sections) and collagen fibers within the pillars were noted in the movie. The movie was created from the stack of sections using Fiji software.

Supplementary movie S6
Three-dimensional reconstruction of SBF- EM transverse sections showing the volume of four intussusceptive pillars. The movie was created using Imaris software Version.7.7.2.
